# Abiotic Stress Effect on *Agastache mexicana* subsp. *mexicana* Yield: Cultivated in Two Contrasting Environments with Organic Nutrition and Artificial Shading

**DOI:** 10.3390/plants13182661

**Published:** 2024-09-23

**Authors:** Judith Morales-Barrera, Juan Reséndiz-Muñoz, Blas Cruz-Lagunas, José Luis Fernández-Muñoz, Flaviano Godínez-Jaimes, Tania de Jesús Adame-Zambrano, Mirna Vázquez-Villamar, Teollincacihuatl Romero-Rosales, María Teresa Zagaceta-Álvarez, Karen Alicia Aguilar-Cruz, Jorge Estrada-Martínez, Miguel Angel Gruintal-Santos

**Affiliations:** 1 Unidad Tuxpan, Facultad de Ciencias Agropecuarias y Ambientales, Universidad Autónoma de Guerrero, km 2.5 Carretera Iguala-Tuxpan, Iguala de la Independencia 40101, Mexico; judith.morales.barrera@gmail.com (J.M.-B.); 18029@uagro.mx (T.R.-R.); 2 Facultad de Ciencias Agropecuarias y Ambientales, Universidad Autónoma de Guerrero, Periférico Poniente s/n, Iguala de la Independencia 40010, Mexico; 01983@uagro.mx; 3 Centro de Investigación en Ciencia Aplicada y Tecnología Avanzada Unidad Legaria, Instituto Politécnico Nacional, Ciudad de Mexico 11500, Mexico; 18414@uagro.mx; 4 Facultad de Matemáticas, Universidad Autónoma de Guerrero, Av. Lázaro Cárdenas s/n, Chilpancingo 39087, Mexico; fgodinezj@uagro.mx; 5 Centro Regional de Educación Superior Campus Zona Norte, Universidad Autónoma de Guerrero, Carretera Taxco-Iguala km 42 s/n, Taxco el Viejo 40323, Mexico; 17021@uagro.mx; 6 Escuela Superior de Ingeniería Mecánica y Eléctrica Unidad Azcapotzalco, Instituto Politécnico Nacional, Av. Las Granjas 682, Col., Alcaldía Azcapotzalco 02550, Mexico; mzagaceta@ipn.mx; 7 Escuela Superior de Ingeniería Mecánica y Eléctrica Unidad Zacatenco, Instituto Politécnico Nacional, Unidad Adolfo López Mateos Av. Luis Enrique Erro s/n, Zacatenco, Gustavo A. Madero 07738, Mexico; kaguilarc@ipn.mx; 8 Centro de Investigación en Petroquímica Secundaria, Instituto Tecnológico de Ciudad Madero, Tecnológico Nacional de Mexico, Prol. Bahía de Aldhair y Av. de las Bahías, Parque de la Pequeña y Mediana Industria, Tecno, Altamira 89440, Mexico; jorge.estrada.mtz@hotmail.com

**Keywords:** abiotic stress, structural parameters analysis, basic cation saturation ratio, isolines, organic nutrition, medicinal and aromatic plants

## Abstract

Research on medicinal plants is essential for their conservation, propagation, resistance to environmental stress, and domestication. The use of organic nutrition has been demonstrated to improve soil fertility and plant quality. It is also important to study the effects of the Basic Cation Saturation Ratio (BCSR) approach, which is a topic where there is currently controversy and limited scientific information. Evaluating the growth and yields of *Agastache mexicana* subsp. *mexicana* (*Amm*) in different environments is crucial for developing effective propagation and domestication strategies. This includes examining warm and subhumid environments with rain in summer in comparison to mild environments with summer rain. Significant differences were observed in the effects of cold, waterlogging, and heat stresses on the plant’s biomass yield and the morphometric-quantitative modeling by means of isolines. The biomass yield was 56% higher in environment one compared to environment two, 19% higher in environment one with organic nutrition, and 48% higher in environment two with organic nutrition compared to using only BCSR nutrition. In the second harvesting cycle, the plants in environment one did not survive, while the plants in environment two managed to survive without needing additional nutrition. Statistical and mathematical analyses provided information about the population or sample. Additionally, further analysis using isolines as a new approach revealed new insights into understanding phenology and growth issues.

## 1. Introduction

The cultivation of medicinal and aromatic plants, such as “purple toronjil” or “Amm”, holds great promise for innovation in the pharmaceutical, food, and cosmetic industries. Thus, the importance of cultivation practices takes high relevance. To enhance the strength and viability of seeds, it is crucial to employ pre-germination treatments, including soaking them in water and subjecting them to biochemical processes such as GA3 or tetrazolium [1,2,3]. Additionally, it is essential to keep the soil well-humidified and improve its physical characteristics to ensure optimal vegetative development and thereby enhance the yield, especially considering that Amm is an undomesticated medicinal plant. Furthermore, the amount of soil organic matter is a key indicator of soil fertility and, therefore, organic farming systems prioritize the use of organic matter to maintain soil health and fertility, aiming to minimize the health risks associated with food production and ensure that the soil’s biological and chemical properties remain intact [4,5]. Crop yield refers to the weight of fresh and dry biomass and the weight and number of harvested seeds. It is influenced by genotype, agronomic management, and environmental conditions. The capability for yield varies among different plant types, depending on their ability to adapt to diverse conditions, including abiotic stresses. In such conditions, it is important to evaluate soil quality and its impact on crop yield by considering the following indicators: (a) the optimal balance of Na, P, Cu, minerals, and organic matter; (b) exploring and combining the relationships and functions among different biological, chemical, and physical parameters; and (c) the correlation among soil chemical composition, Cationic Exchange Capacity (CEC), and physical structure [4,6].

When aromatic plants are exposed to primary abiotic stresses such as low water availability and high salinity, extreme temperatures such as cold or heat stress (low and high temperatures, respectively), hypoxia/anoxia, and nutrient deficiency are the major causes of agricultural production losses. An incorrect combination of these factors and the amount of light, solar radiation, and relative humidity (RH) can affect plant growth and crop yield [7]. High temperatures can harm plants by denaturing proteins and deactivating chloroplast enzymes, increasing water loss through transpiration or evaporation. As a result, the time needed for photosynthesis to produce fruit or seed may be reduced. Also, “the cold stress below 20 °C has a harmful impact on plant growth, development limiting the productivity” and the quality of harvested plants. The affectations can be water intake, metabolic responses, membrane disintegration, and physiological phenomena related to growth and development, besides abnormal anaerobic respiration, leading to the synthesis of abnormal metabolites [8,9].

Without cationic exchange, plants would be unable to obtain sufficient quantities of the essential nutrients to grow, and the nutrients would be leached downward in the soil and lost. Soils with high CEC not only hold more nutrients, fertility and long-term productivity, but they are better able to buffer or avoid rapid changes in soil solution levels of nutrients by replacing them as the solution becomes depleted. The CEC of a particular soil is expressed in units of milliequivalents per 100 g (meq/100 g) of soil. The increase in CEC improved soil structural cohesion and water flow properties [10,11,12]. In a practical approach, the CEC is related to acidity indicators to control Al^3+^ ions saturation in soil. The more CEC (ions different from H^+^ and Al^3+^), the less acidity [13]. Soil balancing is a term that involves applying the Basic Cation Saturation Ratio (BCSR), which assumes that there is an ideal ratio of exchangeable bases, which are mainly cations such as Ca^2+^, Mg^2+^, and K^+^. Under these conditions, the assumptions may be reasonably reliable. If these parameters are absent, then a deficiency in one or more of these nutrients is presumed to exist [14,15]. Base saturation refers to the percent of exchangeable cations on the CEC (meq/100 g soil) and is calculated using the following formula:

$$Base\;saturation\;\left(\%\right)=\left(\frac{total\;bases}{CEC}\right)*100$$

This method needs to take into account several parameters to optimize plant nutrient utilization and crop yield:

(a) Percentage of cations;
(b) Ratios of Ca/Mg, Ca/K, and Mg/K;
(c) A relatively high CEC;
(d) Naturally high soil pH (above 6).

Several studies propose different percentages of an ideal balance of the cations, but this issue can be solved like this: 65–85% of Ca^2+^, 6–20% of Mg^2+^, and 2–5% of K^+^. Additionally, the ideal Ca/Mg, Ca/K, and Mg/K ratios are 6.5/1, 13/1, and 2/1, respectively. The concept of an optimal BCSR is debatable because plant yield and the percentage of the CEC occupied by cations were not always highly correlated in experiments. It should be noted, however, that K, Mg, and Ca concentrations in plants, particularly vegetative parts, were always significantly correlated with BCSR values [14,15,16,17]. 

It has been suggested that incorporating the Balanced Cation Saturation Ratio (BCSR) approach into an overall soil management plan, which includes fertilization, cultural practices, and ensuring nutrient adequacy, is important. To enhance BCSR effectiveness, especially when the Cation Exchange Capacity (CEC) is low, it is essential to emphasize the significance of adding calcium (Ca) to the soil, improving its absorption, considering the entire trace mineral profile, and taking into account the interrelated biological and physical characteristics of the soil [16,18,19,20]. The use of Organic Nutrition (ON) or organic fertilization can greatly improve the physical, chemical, and biological conditions of the soil, such as water-holding capacity to support crop growth and development. A study was conducted on chamomile (*Matricaria chamomilla* L.) to determine the impact of chemical and organic fertilization (compost on soil + liquid compost or leachate). It was found that organic fertilization was more effective than chemical fertilization, as it increased the content of essential oils, the diameter of flower heads, and the dry weight [21]. When the ON content has humic and fulvic acids, consider the following: humic substances are absorbed through plant tissue, raising the amino acid content, nutrients, and vitamins. The indirect effect includes the improvement of the soil’s chemical, physical, and biological properties through water and nutrient retention, ventilation, permeability, greater CEC, root development; also, the direct effects include stimulates biomass accumulation, facilitates mineral nutrient absorption, and increases the resistance of plants to environmental stress [22,23,24,25,26,27]. 

In our efforts to efficiently propagate and domesticate *Amm’s* plants, we undertook: We conducted an experiment on germinating seeds to determine if they were dormant, in order to ensure their growth, studying the impact of abiotic stress in two contrasting environments and examined the interaction effect of the chemical BCSR approach-ON on biomass and fruit yield, considering both quality and quantity. Additionally, throughout the plant’s growth process, we utilized MATLAB software (v9.13, R2022a, The MathWorks Inc., USA) to create three-dimensional maps of the relationships between input and output factors. This approach represents an innovative method in horticulture and complements traditional statistical and mathematical analyses.

## 2. Materials and Methods

### 2.1. Experiment Location and Soil Characteristics

The experimental research was conducted in two different environments located in the northern region of Guerrero, Mexico.

Environment 1 (E1) is situated in the experimental field of the Faculty of Agricultural and Environmental Sciences Unit “Tuxpan”, which is part of the municipality of Iguala de la Independencia in Guerrero, Mexico. Its geographical coordinates are 18°20′38.706″ N and −99°30′6.06″ O, and it stands at an altitude of 735 m above sea level (MASL). The climate is classified as warm and subhumid, with rain in summer (Awo) based on the Köppen classification. The average annual temperature is 26.4 °C, and the annual rainfall is approximately 1100 mm [28].

The soil is of the pelic vertisol type, in color dark brown, with a depth of more than 10 cm, a clay-loam texture and moderate drainage, with 0.536 to 1.47% organic matter, 30% or more clay, 24.16% sand and 41.28% silt, in all its horizons, the pH 7.6 to 8.2, 0.3% of N total, P = 15 ppm, and K = 2 cmol kg^−1^. The soil is not affected by soluble salts or exchangeable sodium [29,30]. 

Environment 2 (E2) is located at the agroecological ranch “El Romerito” in the southeast of the Taxco de Alarcón municipality in Guerrero, Mexico. Its geographical coordinates are 18°35′06.83″ N and −99°40′21.09″ O, and it stands at an altitude of 2334 MASL. The climate is classified as mild, with rain in summer (Cwb) based on the Köppen classification. The average annual temperature is 18.4 °C, and the annual rainfall is approximately 1252 mm [31].

The mountain range is characterized by folded turbidites with abundant fossils from the Late Cretaceous, where a block of foliated rocks consisting of volcanic, terrigenous, and limestone deposits outcrops. The soil type corresponds to a Luvisol, although in the municipality of Taxco, there are also soils classified as Lithosol, Chromic Cambisol, and Haplic Phaeozem [32]. 

### 2.2. Plant Material 

For this research, *Amm* plants were obtained from botanical seeds from previous experimental research at Taxco Guerrero property of the Universidad Autónoma de Guerrero. 

The procedure to obtain plants was as follows:

1. The seeds underwent chemical priming pre-treatment for germination (Citomax^®^), involving a solution with a dose of 1 mL per 1 L of distilled water, and were soaked for 24 h. The Supplementary Materials (see Table S1) provides further details regarding the hormonal components of the germination pre-treatment;
2. The seeds were placed inside a laboratory in sterilized box Petri dishes on a double layer of paper towel moistened with distilled water;
3. Seeds were subsequently watered on their surface by sprinkling until they were covered with a solution of GA3 at 200 ppm (or 200 mg) per liter of water for 4 days until their germination and transformation into seedlings;
4. Once the seedlings had cotyledons (four leaves), they were sown in a tray with 200 cavities in a substrate of Canadian peat and vermiculite mixed in a 3/2 ratio, respectively, until they transformed into plants. During this process, the maximum and minimum temperature and RH were recorded as 33.9 and 28.4 °C and 61.4 and 51.2%, respectively;
5. When the plants reached a height of 8 to 12 cm, they were immersed in a solution of water and rooting agent (at a proportion of 7 g/L) for 5 min. See Table S2 in the Supplementary Materials for further details about the chemical components of rooter treatment.

Note: Not all plants reached these dimensions; to avoid statistical biases, only those that achieved the aforementioned height were selected.

6. They were then transplanted into 15 L polyethylene bags (40 × 40 cm). The substrate was a mixture in a ratio of 50/50 *v*/*v* of humus of leaves and mountain clay loam soil of Taxco de Alarcón municipality. The analysis in the Supplementary Materials (see Table S3) indicates that the soil is loamy;
7. Soil analysis was performed. Refer to the Supplementary Materials for more information;
8. The Supplementary Materials, Figure S7, includes pictures of the seeds, seedlings, and plants.

### 2.3. Experimental Design 

#### 2.3.1. Input Factors and Irrigation

(a) The plants were placed with Artificial Shading (AS) as follows:

In E1, the plants were exposed to direct sunlight from the early hours until noon, when the light was photosynthetically active. The AS was produced because the plants were put under a “mango” tree branches were thinned to avoid direct sunlight during hours of the greatest radiation of the infrared spectrum of sunlight to avoid the plants’ death.

In E2, the AS was from the surrounding trees starting at 2 p.m. and from the clouds typical of the mild environment. 

Note: Our group’s previous studies have demonstrated that AS has a beneficial effect on plant survival.

(b) Four treatments were carried out, two in every environment; where: $\tau_{1}$ = treatment one, $\tau_{2}$ = treatment two, $\tau_{3}$ = treatment three, $\tau_{4}$ = treatment four, as follows:

$$\tau_{1\text{:}}\;E1+\mathrm{AS}+\mathrm{BCSR}\;\mathrm{soil}\;\mathrm{and}\;\tau_{2:}\;E1\;+\;\mathrm{ON}\;+\;\mathrm{AS}\;+\;\mathrm{BCSR}\;\mathrm{soil}$$

$$\tau_{3:}\;E2+\mathrm{AS}+\mathrm{BCSR}\;\mathrm{soil}\;\mathrm{and}\;\tau_{4:}\;E2\;+\;\mathrm{ON}\;+\;\mathrm{AS}\;+\;\mathrm{BCSR}\;\mathrm{soil}$$

**Figure 1:**
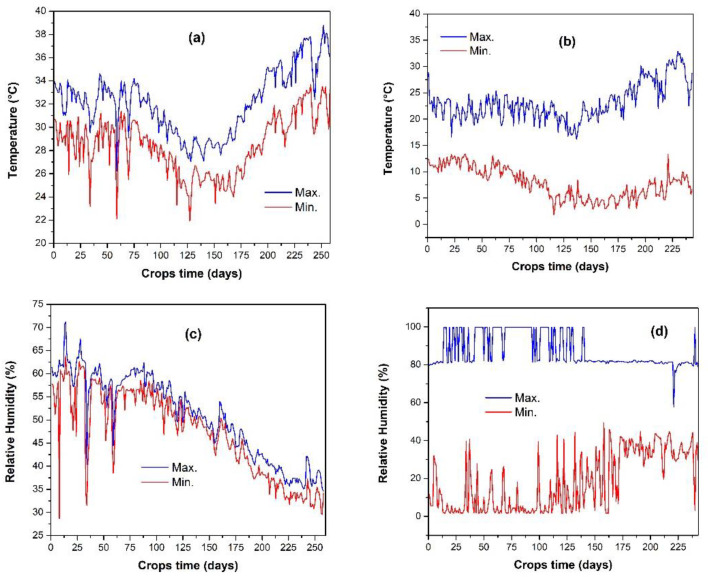
Shows the temperature and relative humidity profiles (maximum and minimum), for both environments: (**a**) Temperature in E1, (**b**) Temperature in E2, (**c**) RH in E1, (**d**) RH in E2.

(c) The Temperature and RH were measured every hour during the crop time with a programmed datalogger instrument in E1 and E2. See the results in Figure 1;
(d) ON = Organic Nutrition or nutrition organic solution. A volumetric solution based on humic and fulvic acids was applied (2 mL/L of water). Note: According to the results of the analysis of the ingredient used for ON (commercial trade Hortihumus^®^), it was considered as mature compost due to: (a) C/N (Carbon/Nitrogen ratio) exceeding 15, (b) the quantity of organic matter, (c) the liquid state, (d) high fulvic and humic acids (12%). See Table S6 in the Supplementary Materials;
(e) I = Irrigation, see Table 1.

Table 1 shows the monthly irrigation profiles during the Crop season. To ensure the plants’ survival, the water quantity had to change over time to avoid the permanent wilting point caused by transpiration. In every environment, the quantity of water was different because temperature and RH were also different.

#### 2.3.2. Output Factors Measure

It is worth noting that the following output factor measurements were performed on the main stem during the Crops time (168 days), 13 times each (all on the same day):

(a) Plant height (H_Pl_) from soil surface until the main stem’s apical meristem with a flexometer;
(b) Leaf number (L_N_);
(c) Internodes number (I_N_);
(d) Branches number (B_N_);
(e) The diameter of the stem (D_T_) was measured with a digital caliper (Vernier trademark SURTEK©, Minimum measuring range—Maximum measuring range = 1 mm–150 mm, Resolution = 0.01 mm) every 10 cm of length until the beginning of the inflorescence, based on the substrate surface. The measurements were made once only for every height in the vegetative development measurement throughout the time;
(f) The Secondary Stem number (SS_N_) emerged from rhizomes

Also noted were:

(a) Inflorescence length (I_L_) was measured with a Truper© FH-8M, Flexmeter Gripper from the first whorl of the inflorescence until the last one (cm);
(b) Chlorophyll content (Ch_C_) (Quantity of chlorophyll content in a leaf). The reading was taken with a chlorophyll meter (Model SPAD-502, Trademark Konica Minolta©, SPAD value: Relative chlorophyll content index; −9.9 to 199.9, difference in optical density at two wavelengths: 650 nm and 940 nm), which quantitatively evaluates the intensity of leaf green always at 01:00 PM, obtaining measurements in three strata of the plant: low, middle and high stratum, in the vegetative and reproductive stages of the plant. The Chlorophyll 1 and 2 content (Ch_C1_, Ch_C2_, respectively) refer to two different measurement dates during the Crops time. Both environments were measured in February (Ch_C1_) and April (Ch_C2_). The reported value in this research corresponds to the middle stratum;
(c) Fresh Weight (W_F_) (biomass of all plants, including roots, before drying);
(d) Dry weight (W_D_) (biomass of all plants, including roots, after the dry process) were weighed on a granite scale model BASE-5EP, Trademark Truper©, Capacity 5 kg, Minimum division 1 g/0.01 oz, Minimum weight 10 g/0.1 oz, measurement units Grams (g)/Ounces (oz).

Note: The dry process was performed at room temperature for seven days inside a laboratory, with temperatures 38.8 and 30.1 °C, and the RH was 37.3% and 30.8% maximum and minimum, respectively. 

#### 2.3.3. Statistical Methodology

In order to determine which treatment yields the best performance based on the output factors, a completely randomized design (CRD) was used to analyze the data. This type of experiment is employed when the experimental units are homogeneous. The treatment comprises four levels, namely τ_1_, τ_2_, τ_3_, τ_4_. The design is balanced, with ten repetitions in each treatment, making it a CRD. The CRD can be expressed as an equation.

$$Y_{ij}=\mu +\tau_{i}+\varepsilon_{ij};\;\;\;\;\;\varepsilon_{ij}\sim IIDN\left(0,\sigma^{2}\right),\;i=1,2,3,4;\;\;\;\;\;n_{1}=n_{2}=n_{3}=n_{4}=10$$

where $Y_{ij}$ is the value of the response variable in treatment i and replication *j*; μ is the global mean of the response variable, $\tau_{i}$ is the effect of treatment i, and the errors $\varepsilon_{ij}$ are supposed to be independent, have a normal distribution with zero mean and constant variance, $\sigma^{2}$. The null hypothesis to test whether there was no effect in terms of treatment in the response variables or equivalently that the populational means of the response variables in the treatments are equal. 

The statistical model assumes normal distribution and constant variance. These assumptions are checked with the Shapiro–Wilks and Bartlett’s tests [33] and car [34] library, respectively, at a significance level of alfa 0.05. However, not all response variables conform to a normal distribution as required by the statistical model. Furthermore, it is not always true that the variances of the response variables of the treatments are equal. If any of the above assumptions fail, then the response variable is transformed using the Box-Cox transformation.

$$y_{\lambda }^{*}=\left\{\begin{array}{c} \frac{y^{\lambda }-1}{\lambda },\;\lambda \neq 0\\ ln\left(y\right),\;\lambda =0 \end{array}\right.$$

The Box-Cox transformation is implemented in the power Transform function of the car library [34] of the R statistical software version 4.0.3 [35]. When the null hypothesis was rejected, the best treatment was identified using Tukey’s test implemented in the *agricolae* Library [36].

#### 2.3.4. Association Analysis of Input and Output Factors Using MATLAB Graphics

Isolines, also known as level curves or contour graphics, represent a function of multiple variables. This type of graphic demonstrates the three-dimensional relationship between two input factors, X and Y (predictors), plotted on the “x” and “y” axis, and the output factors on the “z” axis in various colors. 

**Figure 2:**
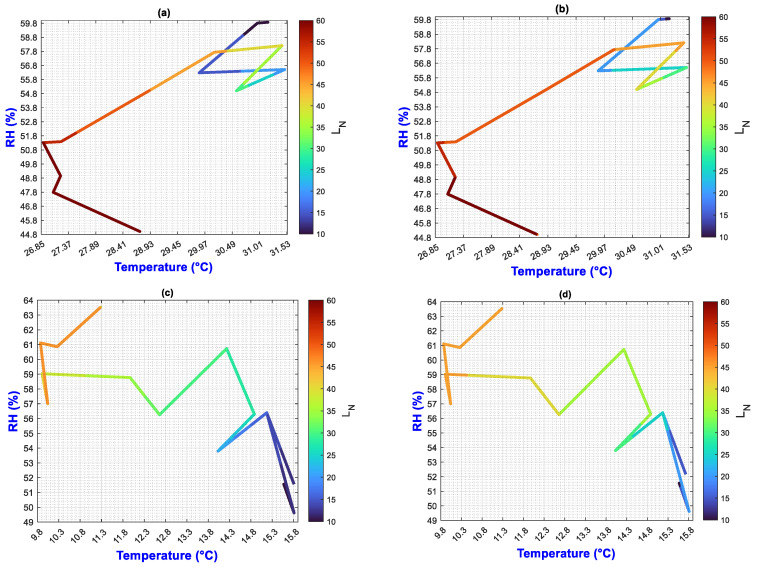
Isolines of L_N_, obtained through relationship RH and Temperature; (**a**) $\tau_{1}$, (**b**) $\tau_{2}$, (**c**) $\tau_{3}$, (**d**) $\tau_{4}$.

**Figure 3:**
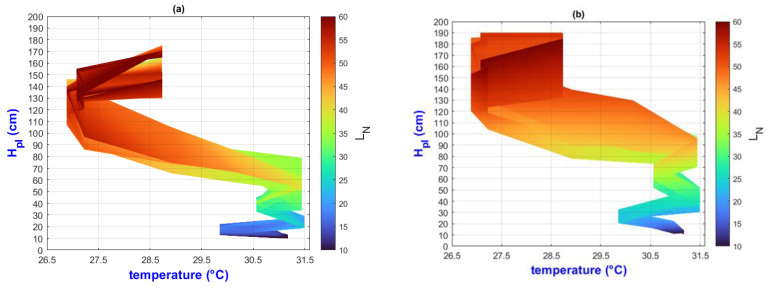

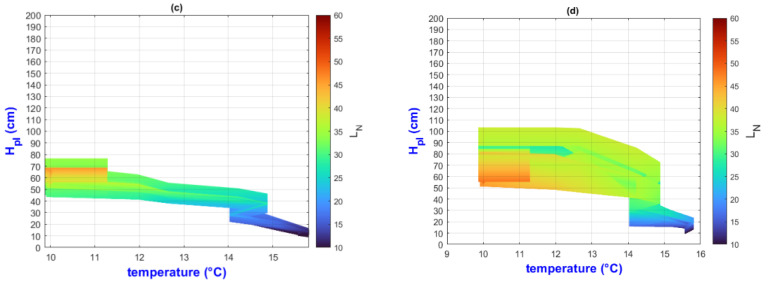
Isolines for relationship among H_Pl_, temperature and L_N_, (**a**) $\tau_{1}$, (**b**) $\tau_{2}$, (**c**) $\tau_{3}$, (**d**) $\tau_{4}$.

**Figure 4:**
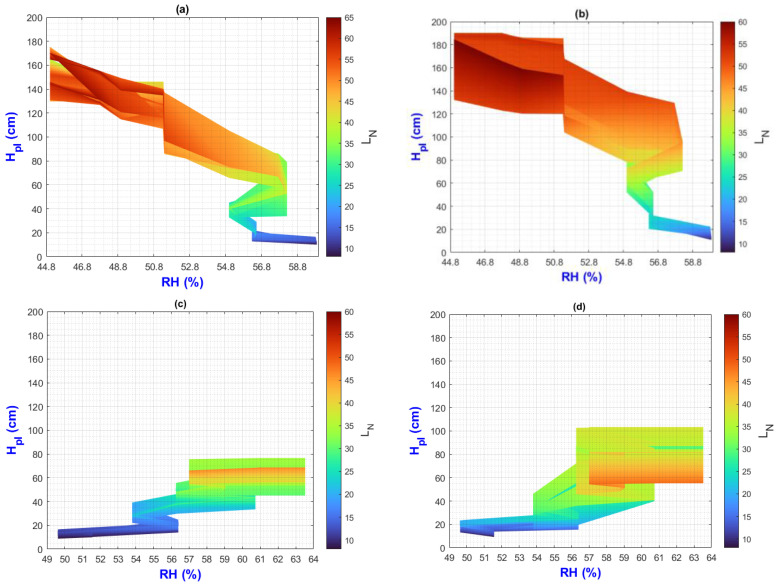
Isolines for relationship among H_Pl_, RH, and L_N_: (**a**) $\tau_{1}$, (**b**) $\tau_{2}$, (**c**) $\tau_{3}$, (**d**) $\tau_{4}$.

**Figure 5:**
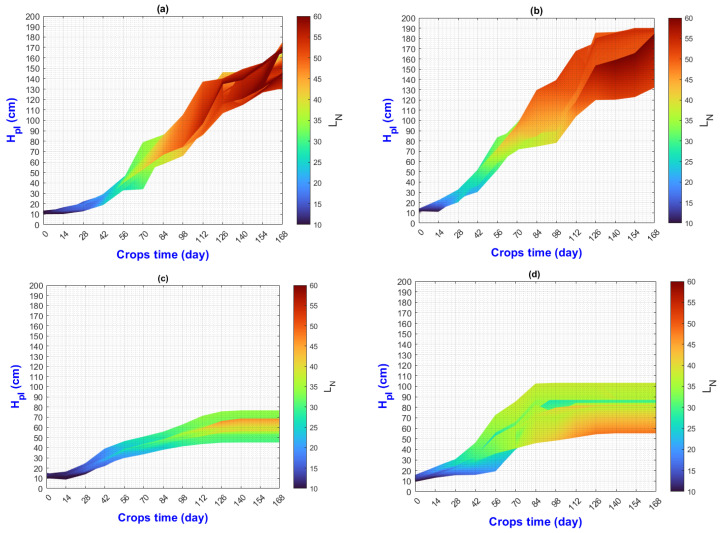
Isolines for relationship among H_Pl_, Crops time, and L_N_: (**a**) $\tau_{1}$, (**b**) $\tau_{2}$, (**c**) $\tau_{3}$, (**d**) $\tau_{4}$.

**Figure 6:**
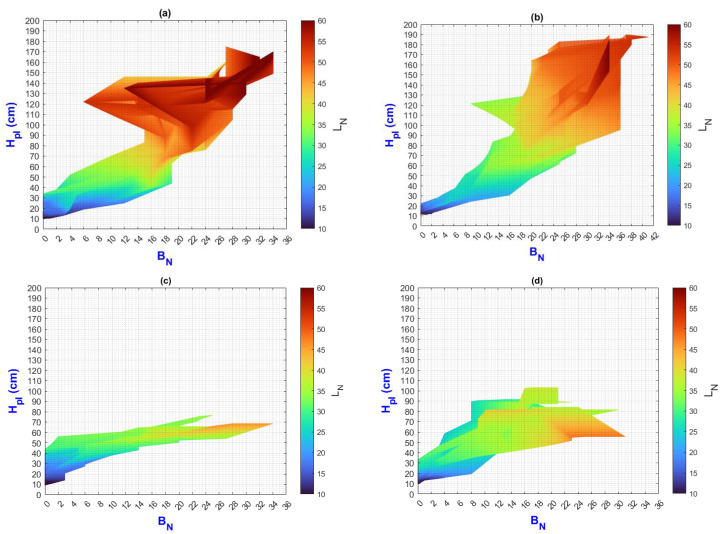
Isolines for relationship among H_Pl_, B_N_, and L_N_: (**a**) $\tau_{1}$, (**b**) $\tau_{2}$, (**c**) $\tau_{3}$, (**d**) $\tau_{4}$.

**Figure 7:**
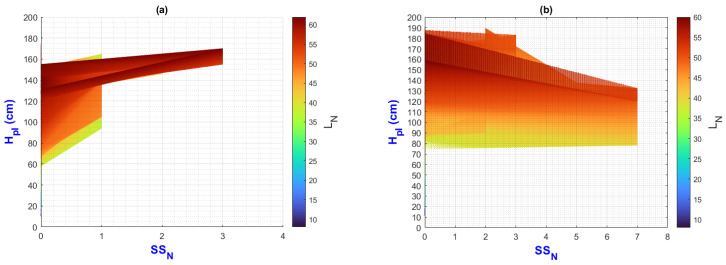

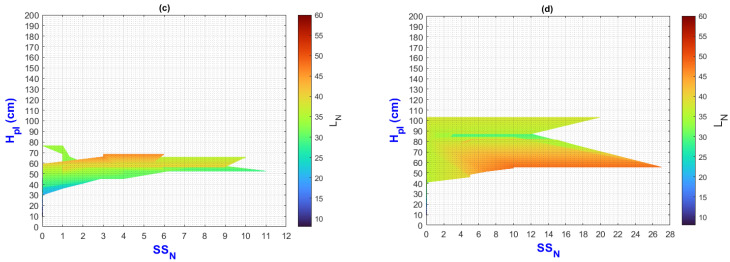
Isolines for relationship among H_Pl_, SS_N_, and L_N_: (**a**) $\tau_{1}$, (**b**) $\tau_{2}$, (**c**) $\tau_{3}$, (**d**) $\tau_{4}$.

**Figure 8:**
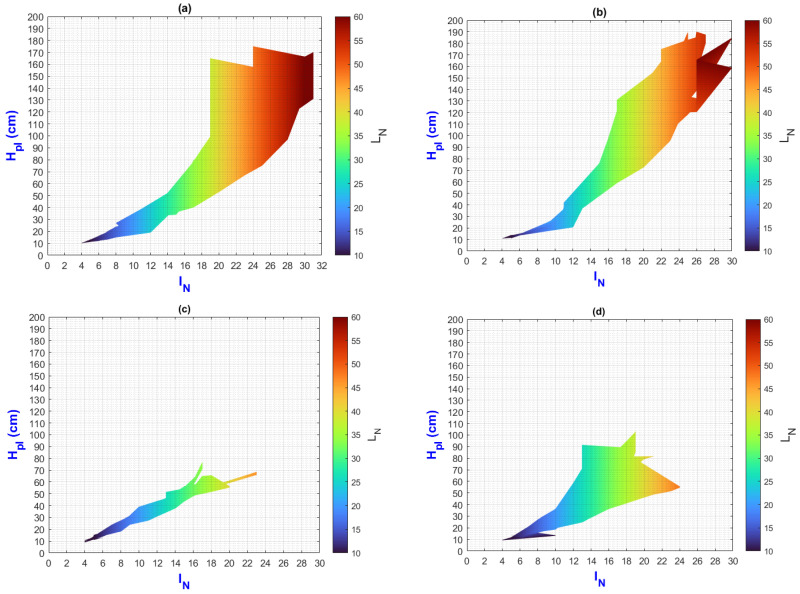
Isolines for relationship among H_Pl_, I_N_, and L_N_: (**a**) $\tau_{1}$, (**b**) $\tau_{2}$, (**c**) $\tau_{3}$, (**d**) $\tau_{4}$.

**Figure 9:**
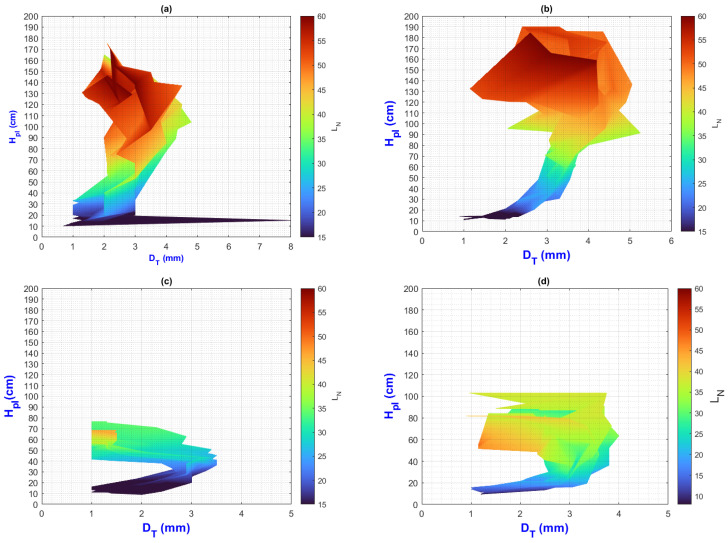
Isolines for relationship among H_Pl_, D_T_, and L_N_: (**a**) $\tau_{1}$, (**b**) $\tau_{2}$, (**c**) $\tau_{3}$, (**d**) $\tau_{4}$.

In this manuscript, the isolines by means of(R2022b) MATLAB’S (v9.13, R2022a, The MathWorks Inc., Natick, MA, USA) Figures in a 3D matrix-graphic arrangement with contour (X, Y, Z, n) are used to obtain the L_N_ isolines as a function of temperature and RH factors where n = 20,000 (isolines analysis to create the image only in Figure 2, Figure 3, Figure 4, Figure 5, Figure 6, Figure 7, Figure 8 and Figure 9). Notice that the output factors of vegetative growth (H_Pl_, I_N,_ B_N,_ D_T,_ SS_N_) are input factors to know their relationship with L_N_. In Figure 2a–d, isolines are only straight because, for every RH, there is only one temperature.

#### 2.3.5. Mathematical Principle Applied in the Plot by Means of MATLAB

We have a hypothetical multivariable function in mathematics $f=f(x_{1},x_{2},x_{3},x_{4},x_{5},x_{6},x_{7,}x_{8,}x_{9})$ that would represent the entire history of the *Amm* plants crop representing the input and output variables where: $x_{1}$ environmental temperature (°C), $x_{2}$ relative humidity (%), $x_{3}$ is the crops time (days); $x_{4}$, is the height of the plant (cm); $x_{5}$ is the number of branches (#); $x_{6}$ is the number of secondary stems (#); $x_{7}$, is the number of internodes (#); $x_{8}$, is the diameter of the stem (mm); $x_{9}$ is the number of leaves (#). Based on the above we can construct the following 3D figures: $f_{1}=f_{1}(x_{1},x_{2},x_{9})$ see Figure 2; $f_{2}=f_{2}(x_{1},x_{4},x_{9})$; see Figure 3; $f_{3}=f_{3}(x_{2},x_{4},x_{9})$ see Figure 4; $f_{4}=f_{4}(x_{3},x_{4},x_{9})$ see Figure 5; $f_{5}=f_{5}(x_{5},x_{4},x_{9})$ see Figure 6; $f_{6}=f_{6}(x_{6},x_{4},x_{9})$ see Figure 7; $f_{7}=f_{7}(x_{7},x_{4},x_{9})$ see Figure 8; $f_{8}=f_{8}(x_{8},x_{4},x_{9})$ see Figure 9, to know its behavior we change the x axis to $x_{1},x_{2},x_{3},x_{5},x_{6},x_{7,}x_{8,}$, We will set the y-axis with $x_{4}$ in all figures, and the z-axis with uppercase $x_{9}$ in all figures. Based on agronomic principles, the height and number of leaves are used as comparative references to analyze their behavior in relation to the plant’s physical parameters.

## 3. Results and Discussion

### 3.1. Statistical Results

Table 2 shows the values of Fresh Weight (g), Dry Weight (g), chlorophyll content (SPAD units), Height of plant (cm), number of leaves, number of nodes, branch number, diameter of stem (mm), and secondary stem number.

Table 3 contains the Box-Cox transformation equations for modeling statistical experimental output factors.

Table 4 shows the sample mean results being very similar in E1 ($\tau_{1}$ and $\tau_{2}$) and very different in E2 ($\tau_{3}$ and $\tau_{4}$). E1 outperformed E2 across all output factors in their values, except for Chlorophyll content and SS_N_.

### 3.2. Environmental Inputs and Agronomic Results Analysis by Means of MATLAB Plots

Figure 1 contains the profile in both environments measured every one hour during the Crops time.

It is important to note that the RH is statistically or completely censored due to low night temperatures. As the temperature drops, the atmosphere becomes saturated with water due to dynamic equilibrium between soil and atmosphere. The difference between the maximum and minimum temperatures and RH is more significant in E2, indicating greater instability. 

The ideal gas law PV = nRT can be used to model the environment before it reaches water saturation. However, in supersaturation, real gas equations are needed. Ions with the same charge, those with a smaller ionic radius, displace those with a greater cationic radius, such as Na^+^, displacing K^+^, Ca^2+^, and Mg^2+^. 

These ions interact with water molecules from the environment and ON, and this interaction is useful in understanding the waterlogging stress on experimental results.

In Figure 2, Figure 3, Figure 4, Figure 5, Figure 6, Figure 7, Figure 8 and Figure 9 (sections a–d), the L_N_ was selected as a reference for vegetative growth, because the leaves are responsible for converting nutrients into biomass through photosynthesis. Understanding the impact of temperature stress originates from the growth elements of the H_Pl_-L_N_ plant and its interaction with other output variables.

Figure 2 L_N_ predictive values were calculated as means of RH and temperature interaction for each measured date throughout the experiment period. 

The next analysis shows how agronomic analysis of vegetative growth can be performed using isolines: 

When isoline lengths change color or direction, then initial and final values of Temperature and RH (and ΔX = ΔT = $T_{2}-T_{1}$ and ΔY = ΔRH = ${RH}_{2}-{RH}_{1}$)

Where $T_{1}$ and $T_{2}$, are the final and the beginning temperatures, also ${RH}_{2}$ and ${RH}_{1}$ are final and beginning relative humidities, which is reflected in the value of L_N_ growth indicated by the color bar or “z” axis. This change is the rate or change ratio. 

The length and slope values of each isoline in Figure 2a–d were calculated using the Euclidean distance formula:

$$d(P_{1},P_{2})=\sqrt{{\left(T_{2}-T_{1}\right)}^{2}+{\left({RH}_{2}-{RH}_{1}\right)}^{2}};\;\mathrm{the}\;\mathrm{slope}\;\mathrm{is}\;m=\frac{{RH}_{2}-{RH}_{1}}{\left(T_{2}-T_{1}\right)}$$

The change ratio in color at each length d of every isoline is different by environment and ON effects. There are 11 and 12 straight intervals to analyze from right to left for treatments $\tau_{1}$-$\tau_{2}$ (Table 5) and $\tau_{3}$-$\tau_{4}$ (Table 6), respectively.

Table 5 and Table 6 show, for every isoline, the values d, m, and initial and final values $L_{N}i$ and $L_{N}f$, respectively. $\left(\frac{L_{N}f}{L_{N}i}\right)$ ratio indicates how much the L_N_ values have increased over time.

An isoline with a longer length and the same slope indicates that the relationship between RH and temperature is more stable over time. This means that fewer factors influence an abrupt change in the environment. 

In E1, vegetative growth reaches its maximum more quickly, while in E2 it is slower. Several events can occur during vegetative development, such as a lack of nutrients, reaching the physiological age necessary to bear fruit, the growth of new secondary stems, thickening of the original stem, or an increase in height. 

These events imply that vegetative development and crop yield continue. Moreover, analyzing the lengths and slopes can indicate the best moments for agronomic management (such as fertilization and pruning), which directly depend on the environment and the physiological phase of the plant. 

A technological application for research can achieve higher crop yields under controlled conditions of temperature and humidity. Then, different zigzag profiles must be designed in relation to lengths and slopes of the isoline.

The following considerations have been made for each treatment in Figure 3 and beyond:

(a) On the “y” axis, it is important to differentiate the difference between final and initial values as ΔY for each point on the “x” axis. Similarly, on the “x” axis, the ΔX should be considered as they represent the behavior of the population, the impact of nutrition, and the environment;
(b) While analyzing ΔX and ΔY, it is important to pay attention to color change (L_N_ values);
(c) The length d of the slope m can be analyzed using the criteria and equations explained in Figure 2;
(d) Pearson’s linear correlation coefficient has been calculated globally for the output factors of all treatments.

These criteria are proposed to analyze isolines, which are a key characteristic in the results.

A more specific analysis of the isolines can be carried out to determine the influence on the growth of H_Pl_ and L_N_; however, for each figure, only a few were performed to show its usefulness.

In Figure 3a–d ($\tau_{1}$–$\tau_{4}$) the isolines show L_N_ profiles by temperature effect. Comparing both environments E1 and E2, it is evident that H_Pl_ and L_N_ are correlated with temperature. The isolines slope, ΔX and ΔY, are greater in E1 than in E2. Section 3.3 analyzes the temperature effect on abiotic stress and other factors in both environments.

In Figure 4a–d ($\tau_{1}$–$\tau_{4}$) the isolines show L_N_ profiles by H_Pl_ and RH effect.

In Figure 4a,b, it is evident that as (RH) decreases, both the height of the plants (H_Pl_) and the number of leaves (L_N_) increase. Specifically, when RH is at 44.8, the plant height increases from 130 to 175 cm (ΔH_Pl_ = 45 cm) at $\tau_{1}$, and from 130 to 190 cm (ΔH_Pl_ = 60 cm) at $\tau_{2}$. This variability suggests a strong impact of the treatment. Notably, the maximum H_Pl_ values do not align with the maximum L_N_ values, potentially indicating the plant’s prioritization of fruit ripening by shedding more leaves. This phenomenon has been observed during drought stress, which will be elaborated on in Section 3.3.

In Figure 4c,d, as the RH increases, H_Pl_ and the L_N_ increase. However, analyzing RH when it increases from 56.5 to 63.5% (ΔRH = 7%), the ΔH_Pl_ of $\tau_{3}$ is lower than the ΔH_Pl_ of $\tau_{4}$. However, the maximum H_Pl_ value does not correspond to the maximum L_N_ value due to the effect of cold stress, which will be explained in Section 3.3.

In summary, in the E1 environment, H_Pl_ and L_N_ vegetative growth occurs as RH decreases from 60% to around 45%. Conversely, in E2, growth happens as RH increases from 50% to around 63%.

In Figure 5a–d ($\tau_{1}$–$\tau_{4}$), the L_N_ isolines represent an ascending sigmoidal profile effect in H_Pl_, which is commonly reported in various publications from all disciplines [37,38]. However, these lines only provide a discrete representation of the population through specific curve fits and/or equations. This means that the sigmoidal profile cannot predict the entire population but only a part of it (See Figure S6 in Supplementary Materials). 

Inside $\tau_{1}$–$\tau_{3}$, the trend is that when H_Pl_ increases, L_N_ also increases. The maximum H_Pl_ values decrease in the following order: $\tau_{2}$_,_ $\tau_{1}$_,_ $\tau_{4}$_,_ $\tau_{3}$, indicating the effect of E1 above E2 and ON above control treatment. Additionally, the growth of H_Pl_ and L_N_ in E2 remained without change for some time. The Pearson’s Linear Correlation Coefficient (PLCC) between H_Pl_ and L_N_ is 0.75 (see Supplementary Materials), reflecting the behavior in $\tau_{4}$, where the maximum L_N_ values correspond to minimum H_Pl_ values (reminder: not all population of plants have this behavior).

In Figure 6a–d ($\tau_{1}$–$\tau_{4}$), when H_Pl_ and B_N_ increase, L_N_ also increases, although ΔB_N_ can increase without needing to increase H_Pl_. The value of BN increases in the following order: $\tau_{4}$, $\tau_{1}$, $\tau_{3},$ and $\tau_{2}$.

Analyzing PLCC values in the B_N_-H_Pl_ and B_N_-L_N_ are 0.57 and 0.68, respectively. Therefore, it can be said that B_N_ has a direct relationship with fresh and dry weight in general.

Note that ΔB_N_ and ΔH_Pl_ (in the whole scale) are greater in E1 than E2.

In Figure 7a, the number of secondary stems increases very slowly when H_pl_ and L_N_ values are maximum. 

In Figure 7b, the maximum values of SSN have the minimum value of H_Pl_ and L_N_. 

In Figure 7c,d, the growth of SS_N_ is inversely proportional to H_pl_.

The PLCC values for H_Pl_-SS_N_ and SS_N_-L_N_ are −0.63 and −0.52, respectively, indicating that vegetative development and the emergence of secondary stems (and perhaps their development) occur alternately.

Figure 8a–d depict a linear relationship where I_N_/L_N_ = 1/2 with varying changes in ΔH_pl_. It is important to emphasize that, for each I_N_ value, there is a corresponding ΔH_pl_, whose value reaches a maximum and then decreases, indicating the end of vegetative development and the start of inflorescence growth for some of the population; meanwhile, a smaller group is still in vegetative development. A possible cause is dormancy in plants.

The PLCC values between I_N_-L_N_ and H_pl_-I_N_ are 1 and 0.75, respectively.

Figure 9a–d illustrate how plants change their diameter in a “mirror C” profile, increasing their H_pl_ to ending their growth with a similar diameter to their initial diameter (the more height, the final diameter moves away from the initial diameter), preparing for blooms and fruit production. 

The PLCC values for H_pl_-D_s_ and D_s_-L_N_ are 0.82 and 0.50, respectively. The plants experienced stress in both environments, and further investigation is needed to determine whether the thickening of the stem is caused by stress, as in the case of *Pinus pinceana Gordon* [39]. 

Remember that this plot represents the vegetative development measurement over time; the final diameter along H_Pl_ until harvest was not measured.

### 3.3. General Discussion 

#### 3.3.1. Germination and Comparison of Yields between Environments

Many medicinal plants have varying degrees of dormancy in their seeds, resulting in uneven germination and subsequent emergence [40]. Our research and other studies by our team have found that pre-treating *Amm* seeds for germination (which typically results in approximately 35% emergence in seedbeds) reveals the characteristic of dormancy in these seeds [41].

Germination began six days after sowing, and approximately 85% of the seeds had germinated after 16 days, demonstrating that the inorganic seed pre-treatment applying GA3 in germination and seedling vigor was successful.

According to our results in *Azospirillum* to ensure good vegetative development, the seed pre-treatment enhanced root growth and weight, which led to increased production of dry leaf, pod, and overall dry matter production in *Cassia angustifolia* [2,5].

In Table 7, the results show the output factors with the highest yields and productivity between environments, corresponding to E1 the favorable trends. Among the E1 treatments, the trend favors $\tau_{2}$. Regarding E2, the trend of higher yields and productivity favors $\tau_{4}$. 

Regarding the previous results, it is relevant to explain that the value of I_L_/H_Pl_ and W_D_/W_F_ ratios of the E1($\frac{\tau_{1}}{\tau_{2}}$) indicates that the plants of both treatments have the same proportion between lengths and weights, being distinguished only by ON. In contrast, in E2, the output factors values and ratios between treatments are smaller, evidencing the effects of temperature, RH, cold stress and waterlogging.

According to research on the germination of ginger (*Zingiber officinale*) and turmeric (*Curcuma longa L*) rhizomes, the optimal temperatures for sprouting were 27.5 °C and 30.1 °C, respectively. Neither species sprouted at 14 °C, and it was found that the minimum temperature required for developing 5 cm shoots was slightly above 17 °C for both. Temperatures above 32 °C caused tissue damage and root loss [42]. Emergence of sugarcane stem pieces showed a linear increase with temperature at 30 °C, and no emergence was observed below 18 °C [43]. These temperature results are relevant to the conditions of E1 and E2 in the production of secondary stems and, in general, on the *Amm’s* vegetative development.

The results of the seed yield are presented in Table 8. Only in E1 were the secondary stems able to produce inflorescence with seed yield. In E2, only the plant with the $\tau_{4}$ was able to produce seed yield; however, these plants also produced the heaviest seeds, which is why the number of seeds per 1 g is the lowest, as a quality indicator. 

Environmental factors influence plant reproductive success. Plants can experience various types of stress, such as water stress, temperature, light, and nutrients, which can be caused by excess or lack of any of these factors. It is important to consider that seed yield occurred later in $\tau_{3}$, indicating that E2, along with the BCSR approach, is more suitable for survival and fruit production. 

#### 3.3.2. Impact of Heat Stress in E1

High-temperature stress can significantly impact cell division, which can restrict plant growth and cause oxidative damage. Even a short exposure to high temperatures during seed filling can lead to low quality and lower yields. High temperatures can be fatal for plants when combined with a limited water supply. The primary cause of water loss due to heat stress is increased transpiration during the day, which can damage critical physiological processes in crops. Heat stress can also reduce root growth, weight, and overall yield, reducing the plant’s access to water and nutrients [44].

In E1, the delay in plants’ flowering and the low is due to a lack of nutrients because of an increment in irrigation to prevent permanent wilting point by heat and drought stresses, reducing the time needed for photosynthesis, among others [8]. However, when the temperature increased, the plant grew, and few secondary stems were produced. At the beginning of the experiment, the temperatures were lower during the rainy season. However, the temperature increased at the start of the year, and RH progressively decreased. 

#### 3.3.3. Impact of Cold Stress in E2

In the E2 soil, abiotic stress was identified with plants’ waterlogging, hypoxia, and cold. This inhibits and/or reduces gas exchange, depriving roots of the oxygen they need for respiration. It also promotes nitrate reduction and affects metabolic conditions, reducing photosynthetic capacity and, thus, plant development yield. In $\tau_{3}$ and $\tau_{4}$ of E2, waterlogging stress is most likely caused by the clay soil conditions, the application of humic and fulvic acids, and the cationic exchange with the environment’s relative humidity, which will be discussed later.

Under cold stress conditions in E2, plants may begin natural torpor (dormancy) because fewer photosynthates are available for translocation to the reproductive areas. As a result, they stop growing, close their stomata, and reduce the availability of sugars in aerial growth meristems. 

Photosynthates move towards the roots or crown, increasing short internode packing due to cold stress. When the soil temperature becomes favorable, rhizomes quickly reproduce and sprout from the surface as secondary stems. In the second phase of photosynthesis, known as the dark phase, plants are classified into three categories: C3, C4, and Crassulacean Acid Metabolism (CAM) plants. C3 plants can survive in very low light conditions but can only partially use solar energy. Maybe our research supports it so that *Amm* can adapt to high temperatures, intense solar radiation, and limited water supply like C4 plants [7].

The environment strongly influences the results in chlorophyll content in Table 2. Therefore, *Amm* plants are more easily adapted to E2 conditions and have more chlorophyll.

Based on their response to low temperatures, plants are categorized into three groups: (a) Chilling-sensitive plants are severely damaged at temperatures above 0 °C and below 15 °C, (b) Chilling-resistant plants sustain injury when ice formation begins in tissues, and (c) Frost-resistant plants can withstand exposure to very low temperatures [9]. *Amm* plants have shown the ability to survive in temperatures between 5 and 15 °C. Therefore, the *Amm’s plants* cannot be classified as chilling delicate plants but as chilling-sensitive plants. 

#### 3.3.4. Impact of BCSR Approach-ON in Yield and Soil Health

*Amm* is a perennial plant that generates new secondary stems from the soil surface after its fruit or seed matures. To observe the new vegetative cycle of these plants in two different environments, they were irrigated after harvested fresh matter and seed. 

The plants in E1 died within three weeks, most likely due to temperature stress, which affected the roots and impacted not only the primary growth cycle but also the secondary growth cycle in woody plants like *Amm* [8]. 

In E2, new secondary stems were developed post-harvest. After 12 weeks, the following central tendency measures were recorded: for $\tau_{3}$, the average was 37.2; the mode was 46, the maximum was 57, and the minimum was 5; for $\tau_{4}$, the average was 65.3, the mode was 80, the maximum was 100, and the minimum was 20. All secondary stems of the plants developed inflorescence and produced seeds. These results reinforced the classification of *Amm* as a chilling-sensitive plant or C3 because cold stress did not affect its quality of life after harvest, in contrast to [8,9]. 

To explain the aforementioned and the results in Table 8, it is necessary to make use of healthy soil issues: this fact is achieved by means of elements and practices for soil regeneration, among them by adding organic matter. “A healthy soil can exhibit the following characteristics: It provides an adequate supply of nutrients to the aboveground biomass, ensuring that plants show no observable symptoms of nutrient deficiency. The soil also has sufficient aggregation of soil particles, allowing water to infiltrate the surface instead of running off. Additionally, it maintains soil organic matter levels relative to pre-row crop agriculture” [45].

The analysis indicates that the ON ingredient used in our experiments is a mature compost, as reflected by its C/N ratio exceeding 15, reflecting its significant humic and fulvic substance. This is an essential factor in determining the variations in the amount of Nitrogen (N) mineralized [5]. Therefore, it is an essential source of organic matter that maintains soil fertility and yield sustainability. 

Researchers observed that BCSR also influences soil structure, particularly surface crusting, compaction, and hydraulic conductivity. The high exchangeable Ca^2+^ content (65%) of a “balanced soil” is beneficial in maintaining and improving soil structure and aggregate stability [18].

According to the soil’s values in our experiment, the base saturation percentages of the cations Ca^2+^, Mg^2+^, and K^+^ in the soil are 78.4%, 19.1%, and 2.01%, respectively. The ratios of Ca/K, Mg/K, (Ca+Mg)/K, and Ca/Mg are 30.9, 9.5, 48.4, and 4.1, respectively. The CEC value is 13.9, which is considered moderately low, and the pH = 6.76, which is considered an optimal value for crops. These ratios and values are consistent with the BCSR approach described in [14,16,45,46]. See Table S3 in the Supplementary Materials.

Then, improving the CEC by adding organic matter is necessary, considering the soil’s characteristics and the effects of fulvic and humic acids.

Loam soil is considered a combination of sand, silt, and clay with ranges of 23–52%, 28–50%, and 7–27%, respectively [47].

Clays and soil organic matter behave as negatively charged ions (anions), allowing them to retain or adsorb cations. This soil capacity enables it to retain the elements necessary to nourish plants [46].

The application of humic substances in soil has been shown to enhance fruit characteristics, diameter, weight, and the number of fruits per plant. Similarly, the use of fulvic acids has been found to increase plant height, leaf number, number of branches and leaves, dry weight, crop growth rate, as well as the number and weight of pods and seed yield, according to the findings of this research [48,49,50]. 

In sandy soils, including loam soils, humic acids bind to sand particles. This increases the CEC and the soil’s capacity to retain moisture and nutrients. As a result, the nutrients are kept in the soil along with water, which makes them accessible to plants. This, in turn, promotes increased plant growth and height [50].

In addition, the values of saturation point, field capacity, and permanent wilting point in the soil of our experiment are 99.8, 53.8, and 32%, respectively (see Table S3 in the Supplementary Materials). These values are very high and, combined with adequate amounts of humic and fulvic acids, ensure that nutrients remain available to the roots as needed, improving structure, fertility, and moisture retention [50]. 

The aforementioned applies to the textural class of loam soils, which retain too much moisture, become compacted in rainy areas, and must be drained. In E2, the pots were not drained, so waterlogging stress occurred [51].

The high CEC in soils enables better retention and utilization of various elements, including minerals and soil nitrogen. This helps to prevent the loss of these compounds through drainage from the root zone. However, in the case of E1, due to heat stress to maintain the field capacity or avoid the permanent wilting point, it was necessary to apply more water irrigation, but this was not enough to achieve better seed weight quality.

Finally, we have included more information about successful researchers on the BCSR approach. See Supplementary Materials.

## 4. Conclusions

According to our research, the use of BCSR soil + ON treatment resulted in higher crop yield compared to the BCSR soil treatment for *Agastache Mexicana* subsp. *mexicana* “Amm” grown in two different environments. Our analysis of the output factors showed that certain factors had an impact on yield, with higher values obtained in E1. The results of crop yield and seed quality were affected by cold, waterlogging, and heat stresses. We also evaluated the crop quality by measuring seed weight and found that the highest values were obtained in $\tau_{4}$ (E2 and ON). The biomass yield between treatments with ON were $\tau_{2}$ is 48% higher than $\tau_{4}$, and control $\tau_{1}$ 66% higher than $\tau_{3}$. Our research confirms the effectiveness of the BCSR approach for achieving higher crop yields, which has been reported by farmers and consultants.

In E1, the Amm plants can be cultivated to produce dry matter as a raw material source for infusions. Future research is necessary to measure terpenes, antioxidants, flavonoids, and others due to heat stress. Seeds can also be produced to improve resistance to extreme environments. Research about propagation by rhizomes, seed vigor and seed germination is necessary. 

In regions where E2 is active, intensive organic cultivation is feasible due to the soil’s natural capacity to generate BCSR and maintain a well-balanced texture. Further research is needed to measure terpenes, antioxidants, flavonoids, and other compounds due to cold stress. Additionally, research on seed germination, seed vigor, and rhizome propagation is needed.

The selection, quantity, proportions, and composition of the ingredients in the experimental soil reflect the knowledge of people living in the mountains. It would be worthwhile to explore the social aspects of the environment as well.

## Figures and Tables

**Table 1:** Irrigation doses during experimental time.

Stage	Total Irrigation per Week			
	$\tau_{1}$	$\tau_{2}$	$\tau_{3}$	$\tau_{4}$
1	3 L water	2 L water + ON	2 L water	1 L water + ON
2	5.5 L water	4.5 L water + ON	2 L water	1 L water + ON
3	7 L water	6 L water + ON	3 L water	2 L water + ON
4	7 L water	6 L water + ON	3.5 L water	2.5 L water + ON

Note 1: For the treatments with ON, one liter of water is prepared as explained in materials and methods. Stage 1: covers the months of August and September, for all treatments the total liters of water are 3 L. Irrigation is applied only on Saturday. Stage 2: covers the months of October, November and December, weekly irrigations were distributed between Wednesday and Saturday in the case of E1 and only on Saturday in the case of E2. Stage 3 and 4 cover the months of January and February, weekly irrigations were distributed between Tuesday, Thursday and Saturday. Note 2: After harvest, the plants in E2 were maintained with only 2.5 L of water per week in the period between the months of May and August.

**Table 2:** Normality and variances homogeneity assumptions results, Box-Cox transformation, and ANOVA results.

Concept		Output Factors *p*-Value									
		W_F_	W_D_	Ch_C1_	Ch_C2_	H_Pl_	L_N_	I_N_	B_N_	D_T_	SS_N_
ANOVA Assumptions	N	0.05	0.08	0.25	0.84	0.48	0.30	0.30	0.91	0.79	0.14
	H	0.21	0.17	0.67	0.95	0.90	0.19	0.19	0.39	0.27	0.57
Box-Cox Parameter	λ	0	0.50	−2.40	7.60	0	--	--	--	−0.30	0.39
R-squared	%	75%	83%	65%	5%	89%	65%	66%	29%	50%	71%
Treatment ANOVA	SSTr	9.55	293.96	1.08 × 10^−8^	1.26 × 10^2^	7.61	2692	673.0	414.88	2.57	172.26
	TMS	3.18	97.99	3.60 × 10^−9^	4.19 × 10^−2^	2.54	897.3	224.3	138.29	0.86	57.42
	$F_{c}(3,\;36)$	36.74	57.55	21.8	0.61	96.05	22.9	22.9	4.84	12.12	29.5
	P	4.7 × 10^−11^	8.2 × 10^−14^	3.1 × 10^−8^	0.61	<2.2 × 10^−16^	1.8 × 10^−8^	1.8 × 10^−8^	6.2 × 10^−3^	1.2 × 10^−5^	8.3 × 10^−10^
Residuals ANOVA	RSS	3.12	61.30	5.93 × 10^−9^	2.50 × 10^−4^	0.95	1410.4	352.6	1028.9	2.55	70.07
	RMS	0.09	1.70	1.65 × 10^−10^	6.83 × 10^−2^	0.026	39.18	9.8	28.58	0.07	1.95

N = Normality (Shapiro–Wilk) H = Homogeneity of variances (Bartlett), TSS Treatment sum of squares, TMS Treatment mean squared, RSS = Residuals sum of squares, RMS Residuals mean squared, A.V. (Analysis of variance), λ = Box-Cox parameter, R-squared = Determination coefficient.

**Table 3:** Box-Cox transformations of the output factors.

T	Box-Cox Transformation						
	W_F_	W_D_	H_Pl_	Ch_c1_	Ch_C2_	D_S_	SS_N_
Ec	$=ln\left(W_{F}\right)$	$=\frac{W_{D}^{0.5}-1}{0.5}$	$=ln\left(H_{Pl}\right)$	$=\frac{{Ch}_{c1}^{-2.397}-1}{-2.397}$	$=\frac{{Ch}_{c2}^{7.599}-1}{7.599}$	$=\frac{D_{T}^{-0.3011}-1}{-0.3011}$	$=\frac{{SS}_{N}^{0.3863}-1}{0.3863}$

**Table 4:** Sample means of the response variables of interest and their Tukey grouping.

$\tau_{i}$	Sample Mean									
	W_F_	W_D_	Ch_C1_	Ch_C2_	H_Pl_	L_N_	I_N_	B_N_	D_T_	SS_N_
O	67.05	20.63	41.7	45.7	118.1	43.8	21.9	25.8	2.0	6.2
$\tau_{1}$	83.1 ^a^	28.5 ^a^	37.3 ^b^	44.6 ^b^	157.6 ^a^	53.2 ^a^	26.6 ^a^	27.3 ^ab^	2.4 ^a^	1.0 ^c^
$\tau_{2}$	103.0 ^a^	34.9 ^a^	35.7 ^b^	46.4 ^b^	170.3 ^a^	50.6 ^a^	25.3 ^a^	30.4 ^a^	2.8 ^a^	2.0 ^c^
$\tau_{3}$	28.2 ^c^	6.3 ^c^	44.8 ^a^	46.1 ^a^	59.8^c^	34.4 ^b^	17.2 ^b^	22.6 ^b^	1.3 ^b^	7.1 ^b^
$\tau_{4}$	53.9 ^b^	12.8 ^b^	49.1 ^a^	45.9 ^a^	84.6^b^	37.0 ^b^	18.5 ^b^	23.0 ^b^	1.5 ^b^	14.8 ^a^

$\tau_{i}$ = Treatment i, O = Overall sample mean. Same letter means no statistically significant difference among the means.

**Table 5:** Values of d, m, of $L_{N}f/L_{N}i$ in E1.

#Isoline/Concept	1	2	3	4	5	6	7	8	9	10	11
d	0.21	3.7	1.65	1.77	3.23	1.45	7	0.62	2.45	1.18	3.23
m	0.3	3.18	0.14	1.64	3.51	0.52	2.17	0.11	7.36	8.36	1.68
$\tau_{1}\;(\frac{L_{N}f}{L_{N}i})$	1	1.6	2.6	3.2	3.8	3.9	4.8	4.8	4.8	4.8	4.8
$\tau_{2}\;(\frac{L_{N}f}{L_{N}i})$	1.6	2.0	2.2	3	3.2	3.4	3.4	4.2	4.4	4.4	4.4

**Table 6:** Values of d, m, of $L_{N}f/L_{N}i$ in E2.

#Isoline/Concept	1	2	3	4	5	6	7	8	9	10	11	12
d	4.85	4.62	2.67	2.83	4.44	4.74	2.60	2.10	2.03	4.11	0.46	2.95
m	12.07	7.16	1.12	2.27	4.62	2.85	3.64	0.13	15.61	24.17	0.64	2.71
$\tau_{3}\;(\frac{L_{N}f}{L_{N}i})$	1.0	1.6	1.2	1.6	2.2	3.0	3.2	3.6	4.0	4.6	4.6	4.6
$\tau_{4}\;(\frac{L_{N}f}{L_{N}i})$	1.3	1.1	1.3	1.4	2.2	2.7	2.8	3.0	3	3	3	3

**Table 7:** Results of output factors, the relationships between treatments, environments, Organic Nutrition, and control treatments.

Yield and Productivity									
Environment	Tr	H_pl_ (cm)	L_N_ (#)	W_F_ (g)	W_D_ (g)	Bloom (%)	I_L_ (cm)	I_L_/H_pl_	W_D_/W_F_
**E1**	$\tau_{1}$	157.6	53.2	83.1	28.5	90	24.5	0.155	0.343
	$\tau_{2}$	170.3	50.6	103.0	34.9	100	26	0.153	0.339
**E2**	$\tau_{3}$	59.8	34.4	28.2	6.3	20	1	0.017	0.223
	$\tau_{4}$	84.62	37.0	53.9	12.8	100	11.3	0.134	0.237
**E1**	$\tau_{1}+\tau_{2}$	319.46	103.8	186.1	63.4	95	50.5	0.308	0.681
**E2**	$\tau_{3}+\tau_{4}$	144.39	71.4	82.1	19.1	60	12.3	0.150	0.460
**E2/E1**	$\frac{{(\tau }_{3}+\tau_{4})}{\left(\tau_{1}+\tau_{2}\right)}$	0.44	0.69	0.44	0.30	63	0.24	0.49	0.68
**Ratios between Treatments**									
**Concept/Output**	**Ratio**	**H_pl_** **(cm)**	**L_N_** **(#)**	**W_F_** **(g)**	**W_D_** **(g)**	**Bloom** **(%)**	**I_L_** **(cm)**	**I_L_/H_pl_**	**W_D_/W_F_**
**E1**	$\frac{\tau_{1}}{\tau_{2}}$	0.93	1.05	0.81	0.82	0.90	0.94	1.01	1.01
**E2**	$\frac{\tau_{3}}{\tau_{4}}$	0.71	0.93	0.52	0.49	0.20	0.09	0.13	0.94
**ON**	$\frac{\tau_{4}}{\tau_{2}}$	0.50	0.73	0.52	0.37	1.00	0.43	0.88	0.70
**BCSR**	$\frac{\tau_{3}}{\tau_{1}}$	0.38	0.65	0.34	0.22	0.22	0.04	0.11	0.65

I_L_ = Inflorescence Length, ON = Organic Nutrition, Tr = treatment. Mathematical ratios are a comparison of the amount of one output contained in another output to understand the impact of environments and treatments. Productivity encompasses the following in outputs: number, lengths, chlorophyll content and percentages.

**Table 8:** Seed characteristics results for $\tau_{1}$, $\tau_{2}$, $\tau_{3}$, $\tau_{4}$.

Concept/ Treatment	MSS (#)	SSS (#)	TS (#)	WMS (mg)	WSS (mg)	TW (mg)	MSSW (mg)	SSSW (mg)	AWpS (mg)	Sp1gr (#)
$\tau_{1}$	94	204	298	5.2	39.0	44.2	0.055	0.191	0.148	5621
$\tau_{2}$	68	626	694	13.9	149.7	163.6	0.204	0.239	0.236	4242
$\tau_{3}$	0	0	0	0	0	0	0	0	0	0
$\tau_{4}$	55	0	55	13.9	0	13.9	0.253	0	0.253	3957

MSS = Main Stem Seeds, SSS = Secondary Stem Seeds, TS = Total Seeds, WMS = Weight Main Stem, WSS = Weight Secondary Stems, TW = Total Weight, MSSW = Main Stem Seeds Weight, SSSW = Secondary Stem Seeds Weight, AWpS = Average Weight per Seed, Sp1gr = Seeds per 1 g.

## Data Availability

The data presented in this study are available on request from the corresponding author.

## Supplementary Materials

The following supporting information can be downloaded at: https://www.mdpi.com/article/10.3390/plants13182661/s1, Soil fertility analysis, physical and chemical properties, Cationic Ratios, Cation Exchange Capacity (CEC) and base saturation percentage of experimental soil, results of ON considered as mature compost, additional analysis to enable the best knowledge, output factors mean, multiple comparison of Tukey, Sample Variance Analysis, and Pictures. Ref. [52] was cited in the Supplementary Materials.

## References

[1] Martínez M.A., Montechiarini N.H., Gosparini C.O., Arango M.R., Gallo CD V., Craviotto R.M. (2019). Viabilidad, vigor y germinación de semillas verdes de soja. Para Mejor. Prod..

[2] Tiwari P., Kumar R. (2020). Effects of pre-sowing seed treatments on germination and seedling growth performance of *Ocimum basilicum* L. J. Pharmacogn. Phytochem..

[3] Mwase W.F., Mvula T. (2011). Effect of seed size and pre-treatment methods of Bauhinia thonningii Schum. on germination and seedling growth. Afr. J. Biotechnol..

[4] Raei Y., Alami-Milani M. (2014). Organic cultivation of medicinal plants: A review. J. Biodivers. Environ. Sci..

[5] Carrubba A. (2015). Sustainable fertilization in medicinal and aromatic plants. Medicinal and Aromatic Plants of the World: Scientific, Production, Commercial and Utilization Aspects.

[6] Karlen D.L., Mausbach M.J., Doran J.W., Cline R.G., Harris R.F., Schuman G.E. (1997). Soil quality: A concept, definition, and framework for evaluation (a guest editorial). Soil Sci. Soc. Am. J..

[7] Ferrante A., Mariani L. (2018). Agronomic management for enhancing plant tolerance to abiotic stresses: High and low values of temperature, light intensity, and relative humidity. Horticulturae.

[8] Husen A., Iqbal M. (2023). Medicinal Plants: Their Response to Abiotic Stress.

[9] Yadav S., Modi P., Dave A., Vijapura A., Patel D., Patel M. (2020). Effect of abiotic stress on crops. Sustain. Crop Prod..

[10] Radulov I., Berbecea A., Sala F., Crista F., Lato A. (2011). Mineral fertilization influence on soil pH, cationic exchange capacity and nutrient content. Res. J. Agric. Sci..

[11] Hodges S.C. (2010). Soil Fertility Basics.

[12] Pernes-Debuyser A., Tessier D. (2004). Soil physical properties affected by long-term fertilization. Eur. J. Soil Sci..

[13] Cruz-Macías W.O., Rodríguez-Larramendi L.A., Salas-Marina M Á., Hernández-García V., Campos-Saldaña R.A., Chávez-Hernández M.H., Gordillo-Curiel A. (2020). Efecto de la materia orgánica y la capacidad de intercambio catiónico en la acidez de suelos cultivados con maíz en dos regiones de Chiapas, México. Terra Latinoam..

[14] Saha N., Mandal B. (2011). Soil testing protocols for organic farming—Concept and approach. Commun. Soil Sci. Plant Anal..

[15] Linder K.J. (2015). The Effect of Soil Cation Balancing on Soil Properties and Weed Communities in an Organic Rotation. Master’s Thesis.

[16] Kopittke P.M., Menzies N.W. (2007). A review of the use of the basic cation saturation ratio and the “ideal” soil. Soil Sci. Soc. Am. J..

[17] Chaganti V.N., Culman S.W., Herms C., Sprunger C.D., Brock C., Leiva Soto A., Doohan D. (2021). Base cation saturation ratios, soil health, and yield in organic field crops. Agron. J..

[18] Zalewska M., Nogalska A., Wierzbowska J. (2018). Effect of basic cation saturation ratios in soil on yield of annual ryegrass (*Lolium multiflorum* L.). J. Elem..

[19] Brock C., Jackson-Smith D., Culman S., Doohan D., Herms C. (2021). Soil balancing within organic farming: Negotiating meanings and boundaries in an alternative agricultural community of practice. Agric. Hum. Values.

[20] Badalingappanavar R., Hanumanthappa M., Veeranna H.K., Kolakar S., Khidrapure G. (2018). Organic fertilizer management in cultivation of medicinal and aromatic crops: A review. J. Pharmacogn. Phytochem..

[21] Hendawy S.F., Khalid K.A. (2011). Effect of chemical and organic fertilizers on yield and essential oil of chamomile flower heads. Med. Aromat. Plant Sci. Biotechnol..

[22] Tan K.H. (2003). Humic Matter in Soil and the Environment: Principles and Controversies.

[23] Melo López L. Análisis y caracterización de ácidos fúlvicos y su interacción con algunos metales pesados. 2006.

[24] Cooper L., Abi-Ghanem R. (2017). El Valor de las Sustancias Húmicas en el Ciclo de Vida del Carbón de los Cultivos: Ácidos Húmicos, ácidos Fúlvicos.

[25] Veobides-Amador H., Guridi-Izquierdo F., Vázquez-Padrón V. (2018). Las sustancias húmicas como bioestimulantes de plantas bajo condiciones de estrés ambiental. Cultiv. Trop..

[26] Haghighi M., Kafi M., Colmillo P. (2012). Actividad fotosintética y metabolismo de N de la lechuga afectados por el ácido húmico. Rev. Int. Cienc. Veg..

[27] Canellas L.P., Olivares F.L., Aguiar N.O., Jones D.L., Nebbioso A., Mazzei P., Piccolo A. (2015). Humic and fulvic acids as biostimulants in horticulture. Sci. Hortic..

[28] García E. (2005). Modificaciones al Sistema de Clasificación Climática de Köppen.

[29] Alcántara Jiménez J.Á., Hernandez Castro E., Ayvar Serna S., Nava A.D., Brito Guadarrama T. (2010). Phenotypic and agronomic characteristics of six genotypes of papaya (*Carica papaya* L.) from Tuxpan, Guerrero, Mexico. Rev. Venez. Cienc. Tecnol. Aliment..

[30] Damián-Nava A., González-Hernández V.A., Sánchez-García P., Peña-Valdivia C.B., Livera-Munoz M., Brito-Guadarrama T. (2004). Crecimiento y fenología del guayabo (*Psidium guajava* L.) cv.“Media China” en Iguala, Guerrero. Rev. Fitotec. Mex..

[31] García E. (2004). Modificaciones al Sistema de Clasificación Climática de Köppen.

[32] Chávez-Hernández C.G., Barrera Aguilar C.C., Téllez Espinosa G.J., Chimal-Sánchez E., García-Sánchez R. (2021). Colonización micorrízica y comunidades de hongos micorrizógenos arbusculares en plantas medicinales del bosque templado “Agua Escondida”, Taxco, Guerrero, México. Sci. Fungorum.

[33] Gross J., Ligges U. (2015). Nortest: Tests for Normality. R Package Version 1.0-4. https://cran.r-project.org/web/packages/nortest/.

[34] Fox J., Weisberg S. (2019). An R Companion to Applied Regression.

[35] R Core Team (2020). R: A Language and Environment for Statistical Computing.

[36] Felipe de Mendiburu (2021). Agricolae: Statistical Procedures for Agricultural Research. R Package Version 1.3-5. https://cran.r-project.org/web/packages/agricolae/index.html.

[37] Longhi D.A., Dalcanton F., Aragão G.M.F.D., Carciofi B.A.M., Laurindo J.B. (2017). Microbial growth models: A general mathematical approach to obtain μ max and λ parameters from sigmoidal empirical primary models. Braz. J. Chem. Eng..

[38] Yuan R., Yang B., Liu Y., Huang L. (2019). Modified Gompertz sigmoidal model removing fine-ending of grain-size distribution. Open Geosci..

[39] Martiñón-Martínez R.J., Vargas-Hernández J., López-Upton J., Gómez-Guerrero A., Vaquera-Huerta H. (2010). Respuesta de Pinus pinceana Gordon a estrés por sequía y altas temperaturas. Rev. Fitotec. Mex..

[40] Saeedeh R., Mehrnaz H., Mansour G. (2021). Silicon-nanoparticle mediated changes in seed germination and vigor index of marigold (*Calendula officinalis* L.) compared to silicate under PEG-induced drought stress. Gesunde Pflanz..

[41] Reséndiz-Muñoz J., Cruz-Lagunas B., Fernández-Muñoz J.L., de Jesús Adame-Zambrano T., Delgado-Núñez E.J., Zagaceta-Álvarez M.T., Aguilar-Cruz K.A., Urbieta-Parrazales R., Miranda-Viramontes I., Morales-Barrera J. (2023). Influence of artificial shading and SiO2 on *Agastache mexicana* subsp. *mexicana* Ability to survive under water stress. Horticulturae.

[42] Retana-Cordero M., Fisher P.R., Gómez C. (2021). Modeling the effect of temperature on ginger and turmeric rhizome sprouting. Agronomy.

[43] Smit M.A. (2011). Characterising the factors that affect germination and emergence in sugarcane. Int. Sugar J..

[44] Passioura J.B. (1983). Roots and drought resistance. Developments in Agricultural and Managed Forest Ecology.

[45] Dougherty B. (2019). Regenerative Agriculture: The Path to Healing Agroecosystems and Feeding the World in the 21st Century.

[46] Garrido Valero M. (1994). Interpretación de Análisis de Suelos. https://www.mapa.gob.es/ministerio/pags/biblioteca/hojas/hd_1993_05.pdf.

[47] FAO (2019). Organización de las Naciones Unidas Para la Alimentación y Agricultura. Textura del Suelo. https://www.fao.org/fishery/docs/CDrom/FAO_Training/FAO_Training/General/x6706s/x6706s06.htm.

[48] Yildirim E. (2007). Foliar and soil fertilization of humic acid affect productivity and quality of tomato. Acta Agric. Scand. Sect. B-Soil Plant Sci..

[49] Abdel-Baky Y.R., Abouziena H.F., Amin A.A., Rashad El-Sh M., Abd El-Sttar A.M. (2019). Improve quality and productivity of some faba bean cultivars with foliar application of fulvic acid. Bull. Natl. Res. Cent..

[50] Varas Abad P.L. (2012). Evaluación de Dosis de Ácido Húmico Granulado de Leonardita y Ácidos Húmicos y Fúlvicos con Macro y Micro Nutrientes en el Cultivo de Cebollita China (Var. Roja chiclayana), Bajo Condiciones Agroecológicas en la Provincia de Lamas.

[51] https://agrawdata.com/blog/suelos-francos/.

[52] Blevins R.L., Frye W.W. (1993). Conservation tillage: An ecological approach to soil management. Adv. Agron..

