# Supplementary material for: Abiotic Stress Effect on Agastache mexicana subsp. mexicana Yield: Cultivated in Two Contrasting Environments with Organic Nutrition and Artificial Shading
Source: Plants (Basel). 2024 Sep 23;13(18):2661. doi: 10.3390/plants13182661 (PMC11435050; doi:10.3390/plants13182661)
Supplement: Supplementary file 1 [file plants-13-02661-s001.zip › plants-3169373-supplementary.pdf]

Table S1. Components of hormonal chemical priming treatment

Hormonal treatment	
Component	Units
Cytokinins	6,000 ppm
Auxins	100 ppm
Fulvic Acids	2.20 %
Chelating Agents (Ca)	4.32 %
Phosphorus	1,000 ppm
Nitrogen	4.0 %

Table S2. Chemical components of Rooter treatment

Rooter content	
Component	Unit
Total Nitrogen	16 %
Available Phosphorus	45 %
Potassium	11 %
Magnesium	0.6 %
Zn	0.4 %
B	0.2 %
Sulfur	0.8 %
Phytohormones	400 ppm

Table S3. Soil fertility analysis, physical, chemical properties

Soil´s textural class: Loamy

**Determination**	**Result**	**Unit**
Saturation point	99.8	%
Field capacity	53.8	%
Permanent wilting point	32.0	%
Hydraulic condition	0.1	% (cm/h)
Apparent density	0.6	g/cm^3
pH (1:2 water)	6.76	neutral
Total carbonates	0.01	%
Salinity (EC)	0.73	dS/m
OM	21.7	%
P	3.81	ppm
K	110	ppm
Ca	2193	ppm

Mg	323	ppm
Na	<25	ppm
Fe	24.3	ppm
Zn	2.11	ppm
Mn	25.6	ppm
Cu	0.35	ppm
B	0.4	ppm
S	0.10	ppm
$N(NO_3)^-$	30.7	ppm

OM = Organic Material, ppm = parts per million,

Table S4. Cationic Ratios

Ratio between cations (based on me/100g)				
Ratio	Ca:K	Mg:K	(Ca+Mg):K	Ca:Mg
Result	38.9	9.5	48.4	4.10

Table S5. Cation Exchange Capacity (CEC) and base saturation percentage

Exchangeable cations					
Sat (%)	78.4	19.1	2.01	0.07	
me/100 g	10.9	2.66	0.28	0.01	13.9
Cation	Ca^{2+}	Mg^{2+}	K^+	Na	CEC

Table S6. Results of ingredient for Organic Nutrition (Hortihumus®)

**Analysis**		
**Determination**	**Result**	**NCC**
pH	11.7	
Electrical conductivity	68.9	
Total Nitrogen (N)	0.29 %	3 g/L
* Phosphorus (P)	0.0030 %	0 g/L
* Potassium (K)	2.51 %	30 g/L
* Calcium (Ca)	0.05 %	1 g/L
* Magnesium (Mg)	0.06 %	1 g/L
* Sodium (Na)	1.29 %	13 g/L
** Sulfur (S)	1.02 %	10 g/L
** Iron (Fe)	390 ppm	390 mg/L
** Copper (Cu)	0.59 ppm	1 mg/L
** Manganese (Mn)	29.0 ppm	29 mg/L

** Zinc (Zn)	2.52 ppm	3 mg/L
** Boron (B)	16.9 ppm	17 mg/L
Moisture	69.6 %	
Organic material	12.5 %	
Ashes	17.8 %	
Organic Carbon	7.27 %	
C/N Ratio	25.1	

*Micronutrients, ** Macronutrients, NCC= Nutrient Contribution of Compost

Note: the laboratory considers that this ingredient is a madure compost by its nature.

This section has been included because we consider that horticultural and scientific communities can complement their interpretation of the results

Figure S1. The relationships among H_{Pl}, L_N and Crops Time in 3D, (a) τ_1, (b) τ_2, (c) τ_3, (d) τ_4

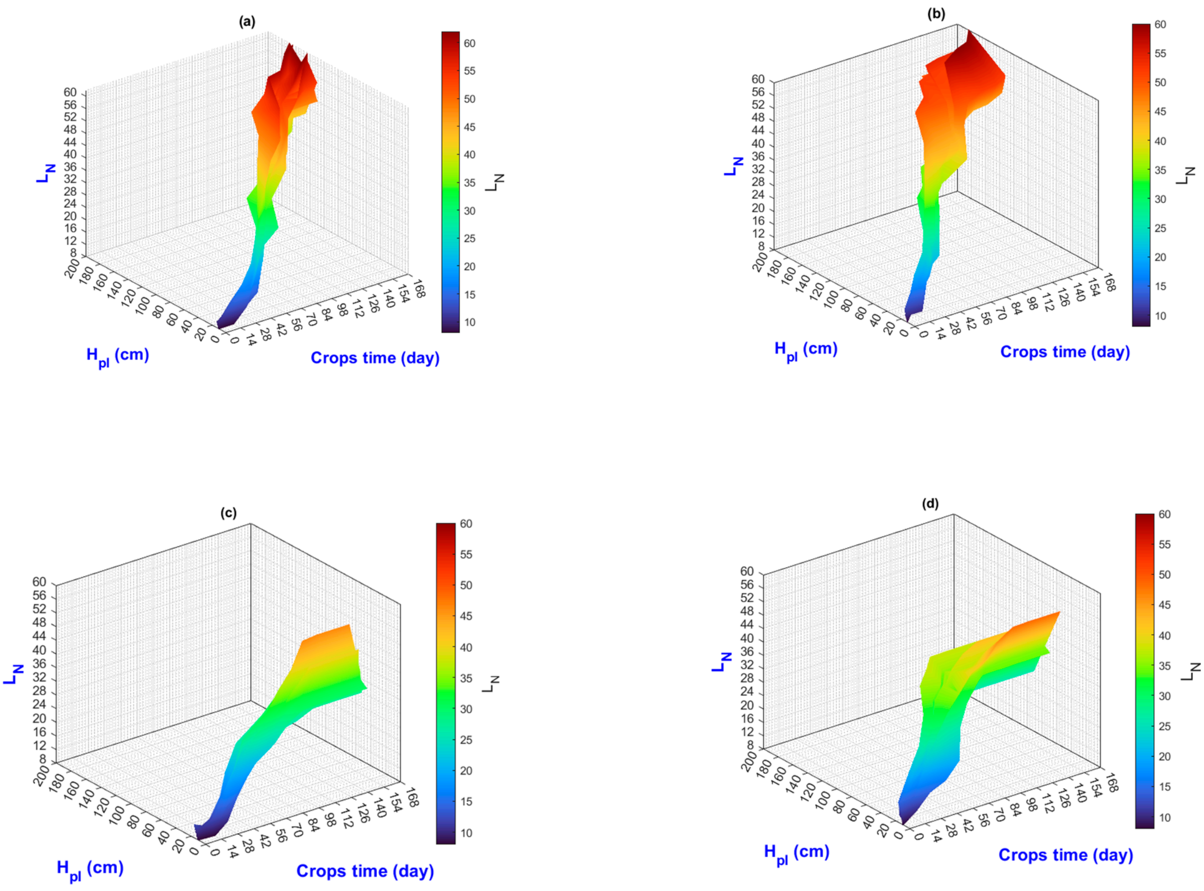

Figure S2. The relationships among H_{Pl}, L_N and D_T in 3D, (a) τ_1, (b) τ_2, (c) τ_3, (d) τ_4

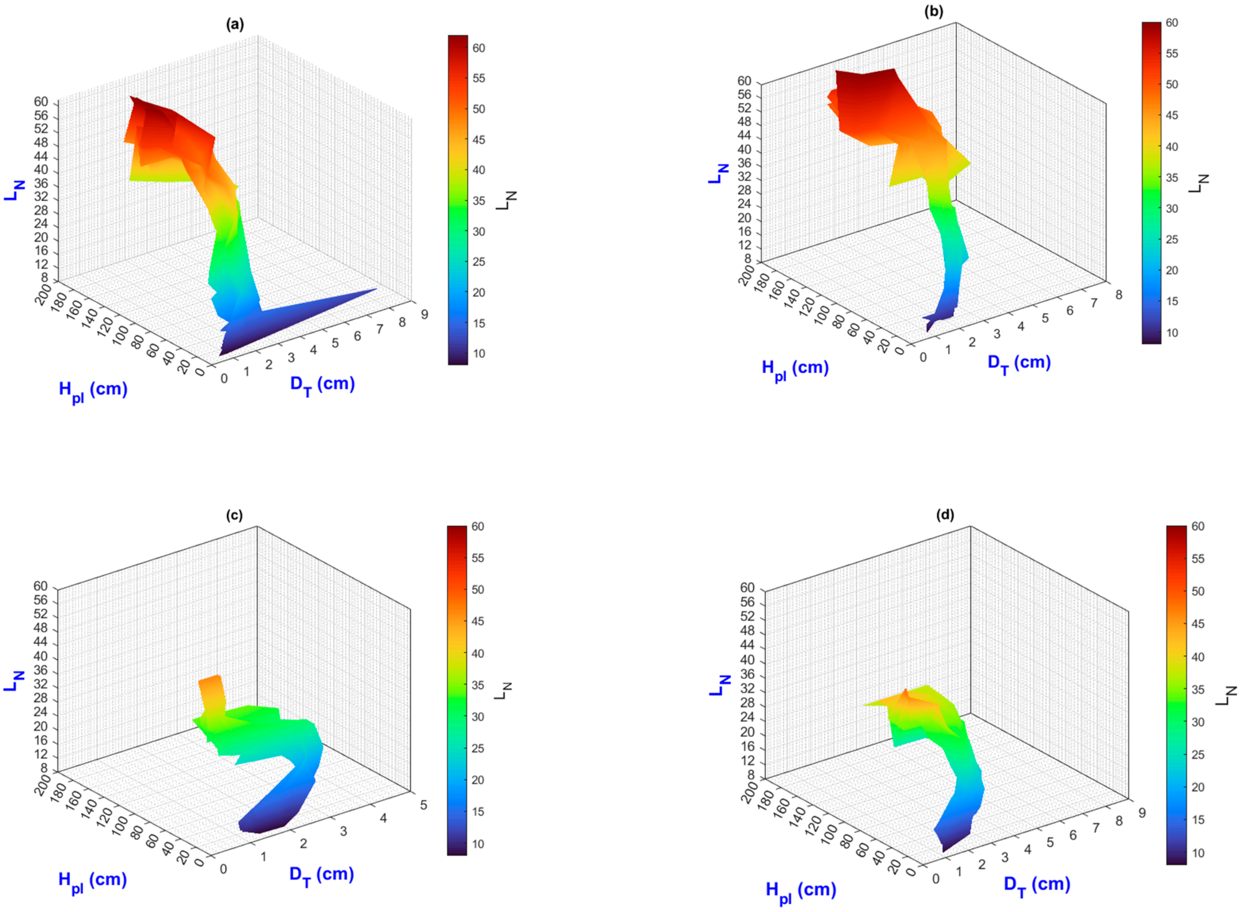

Figure S3. The relationships among H_{Pl}, L_N and B_N in 3D, (a) τ_1, (b) τ_2, (c) τ_3, (d) τ_4

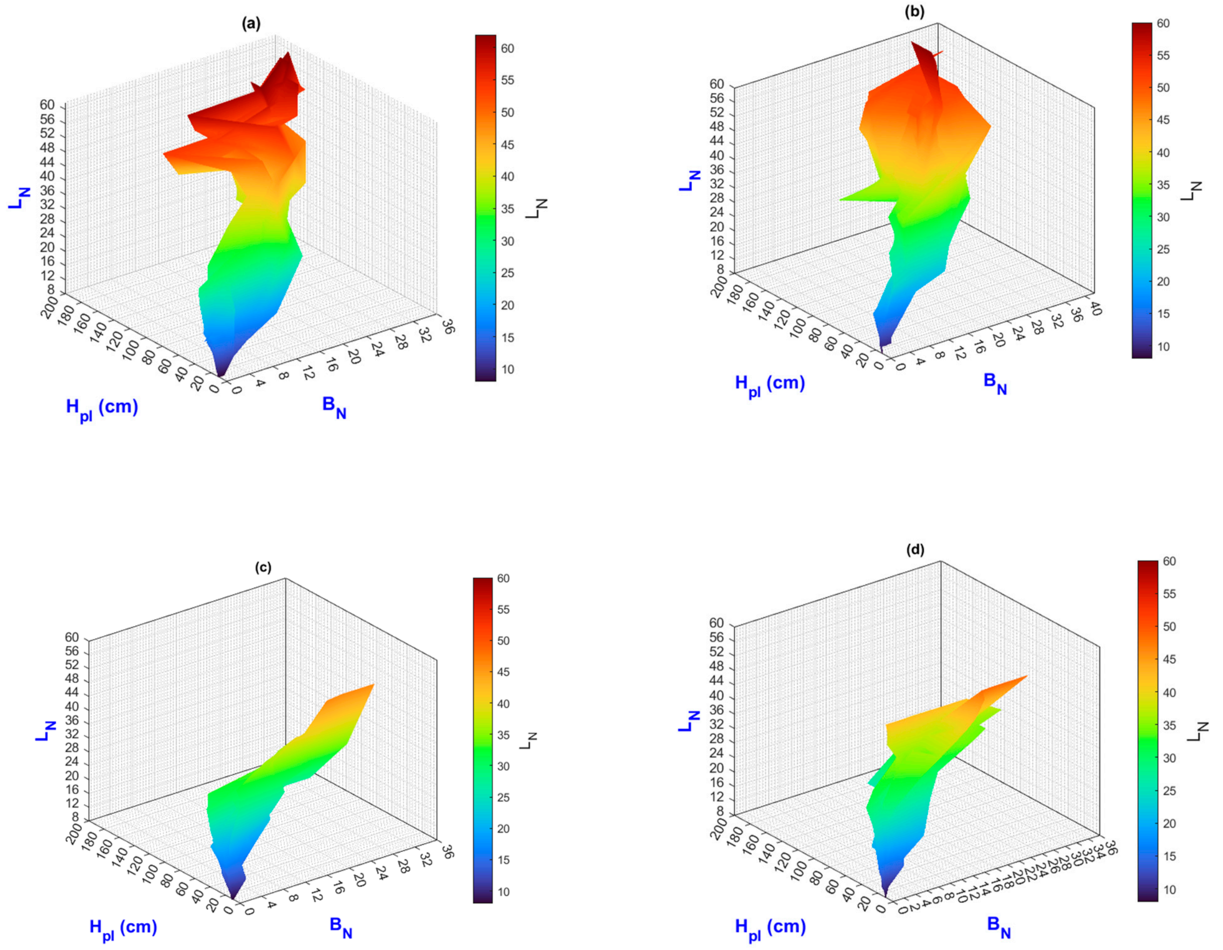

Figure S4. The relationships among H_{Pl}, L_N and SS_N in 3D, (a) τ_1, (b) τ_2, (c) τ_3, (d) τ_4

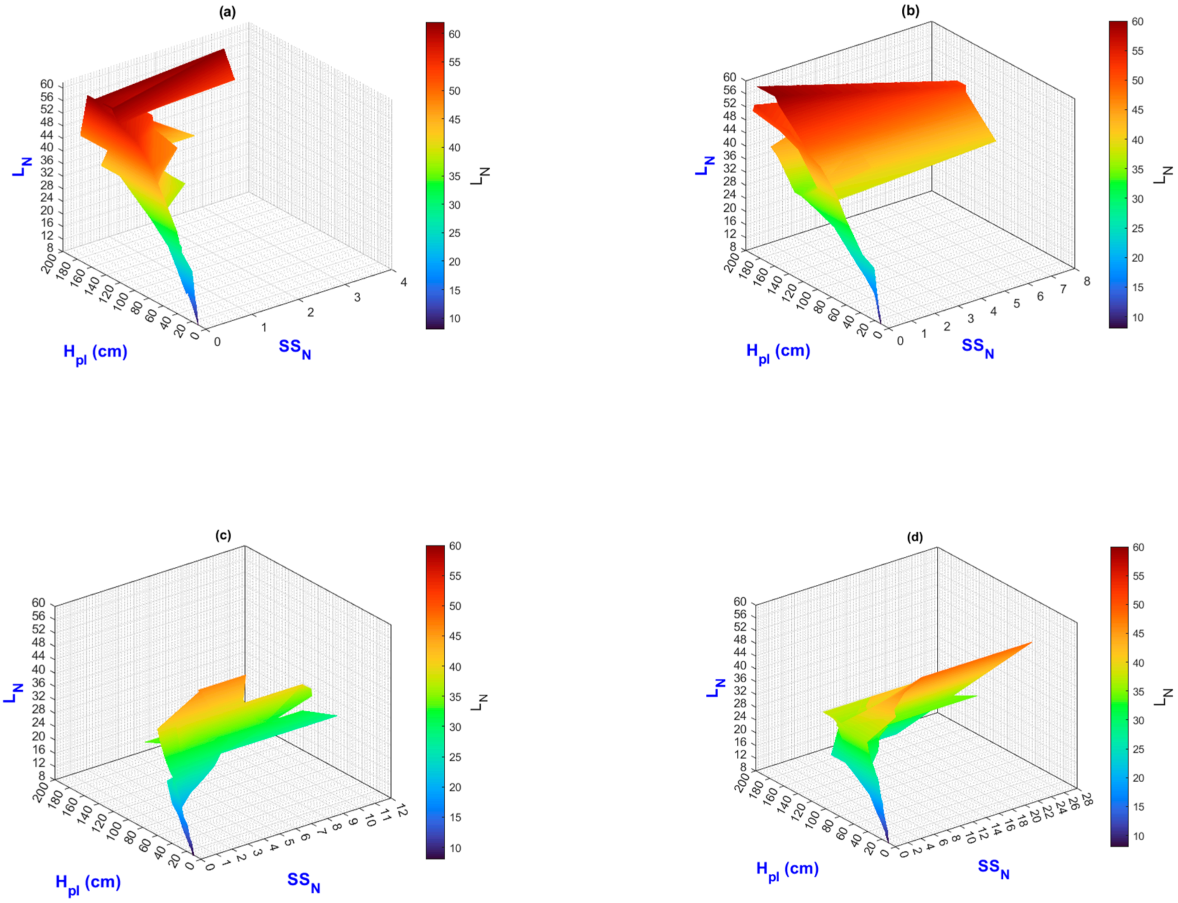

Figure S5. The relationships among H_{Pl}, L_N and I_N in 3D, (a) τ_1, (b) τ_2, (c) τ_3, (d) τ_4

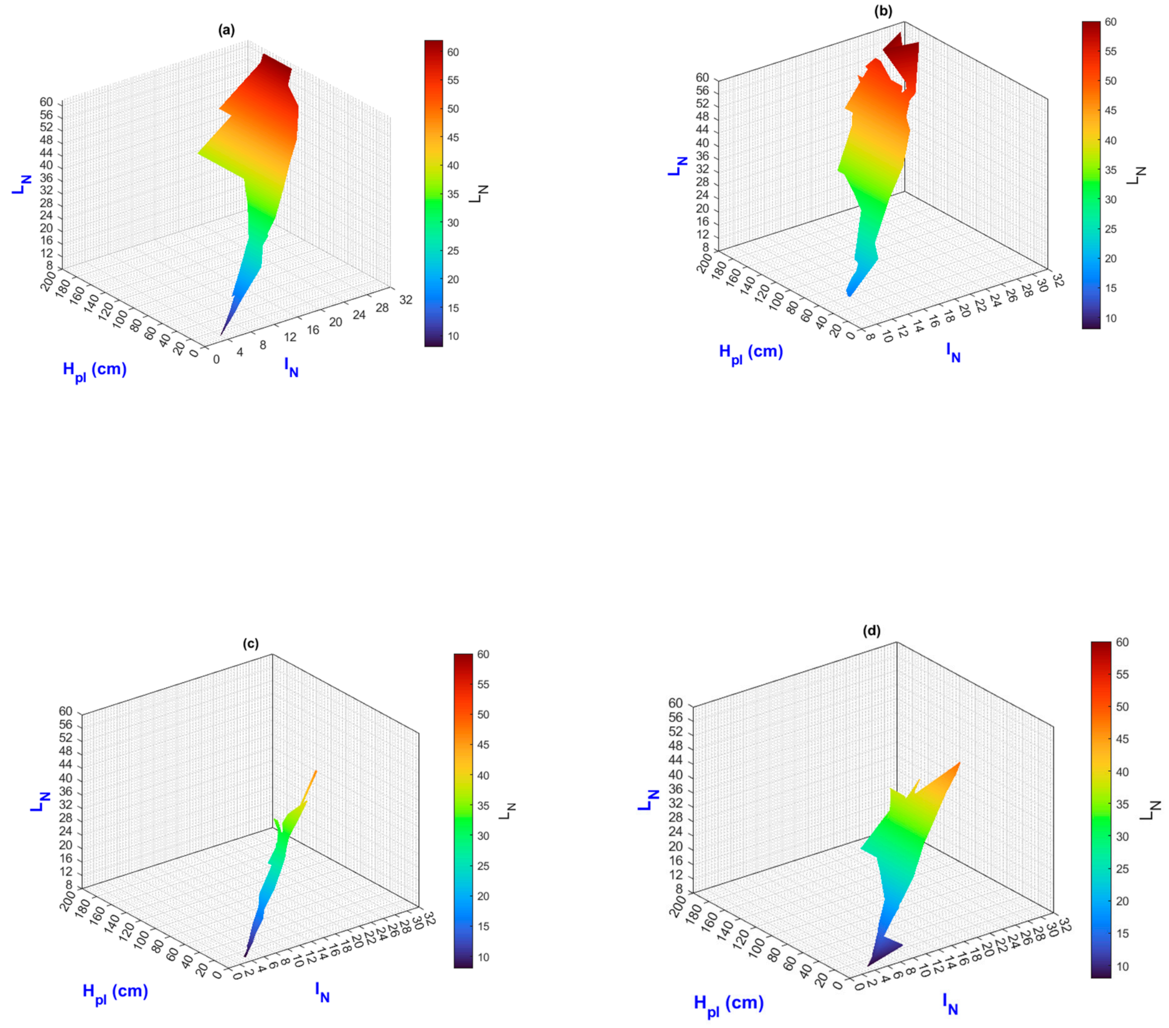

Figure S6. The plot depicts graphs a-l that effectively summarize the experimental results of treatments τ_1 -τ_4. It accurately adjusts sigmoidal trends, growth potentials, straight lines, interpolation, and grouping over Crops time. The provided information specifically relates to τ_2.

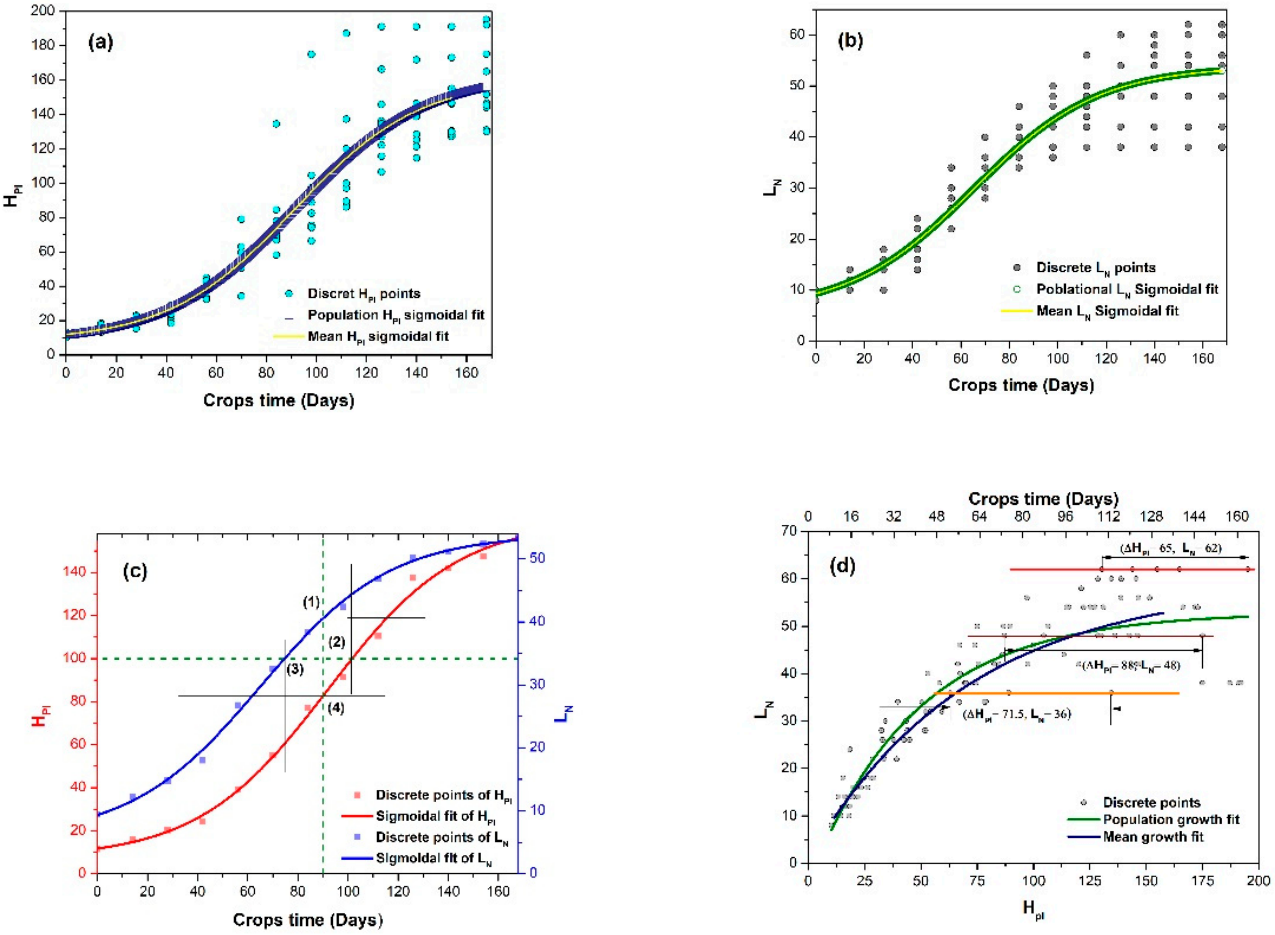

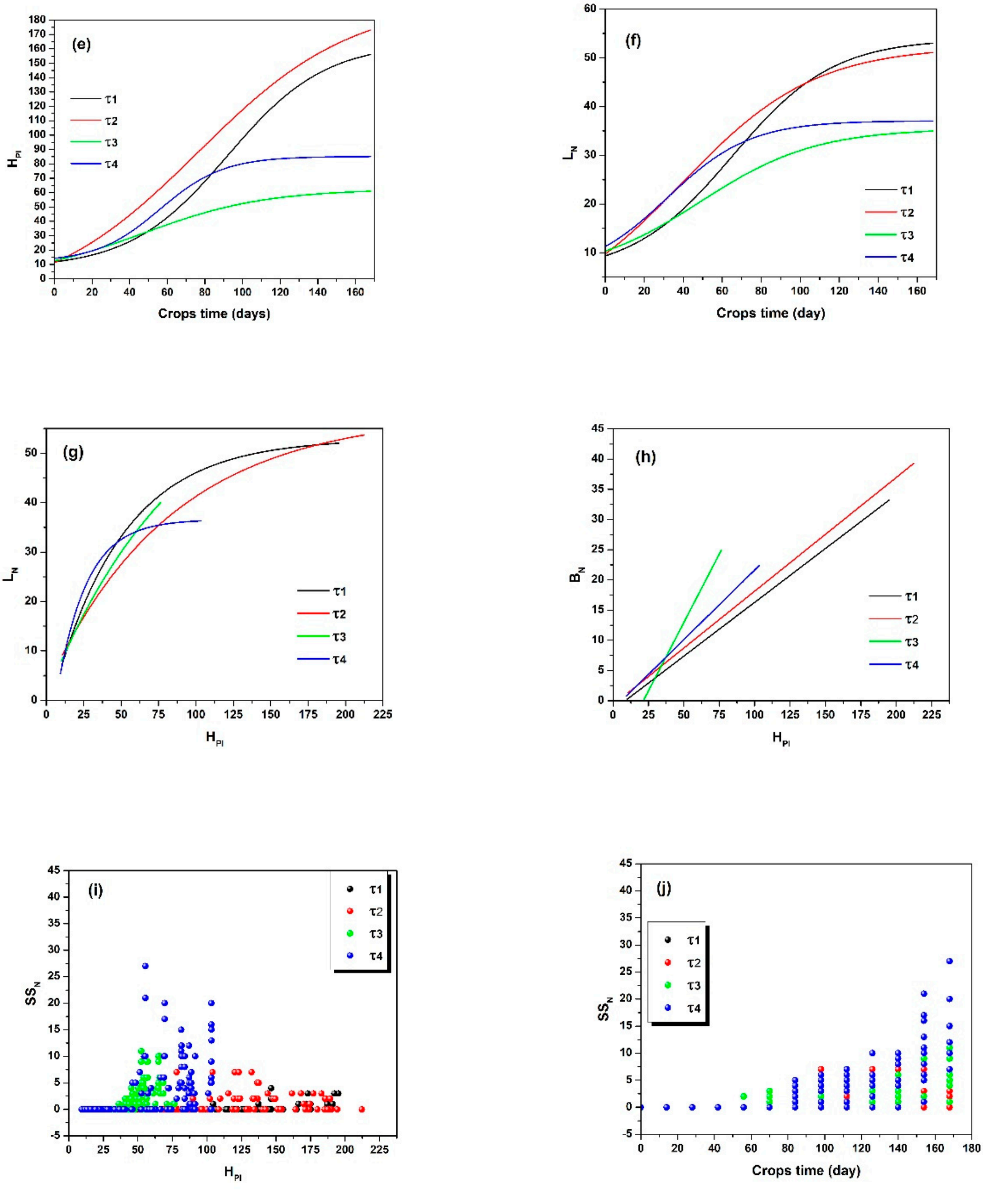
(e)
H_PI
Crops time (days)
τ1
τ2
τ3
τ4
(f)
L_N
Crops time (day)
(g)
L_N
H_PI
(h)
B_N
H_PI
(i)
SS_N
H_PI
(j)
SS_N
Crops time (day)

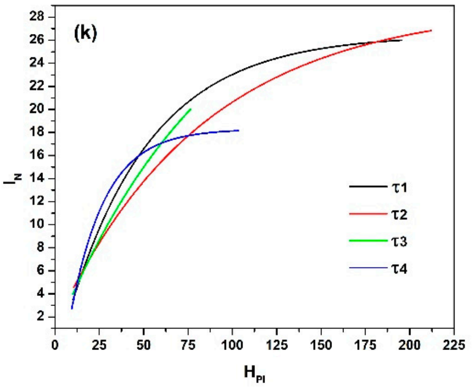

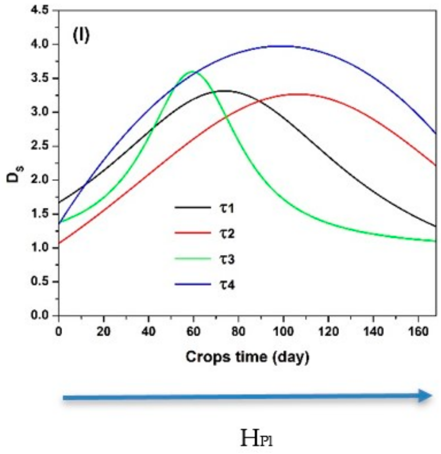

Figure S6a and S6b show the sample mean and full sample sigmoidal fits of H_{PI} and L_N respectively, which are very similar and superposed.

The graph in Figure S6c displays a sigmoidal correlation between the time spent on Crops (x-axis) and the average data of H_{PI} and L_N (y-axis). By drawing a vertical and horizontal solid line that intersects with the dashed lines (points 1-4), we can obtain the values of H_{PI} and L_N for each Crops time. However, we can only gather this information from the points of intersection between the solid and dashed lines, not from the intersection of the solid lines themselves.

Figure S6d displays a correlation between H_{PI} and L_N with the exponential fit shown on the graph. The horizontal lines in red, wine, and orange represent discrete points at L_N, with ΔH_{PI} (initial and final points on the x-axis) shown as ($x = \Delta H_{PI}$, $y = L_N$). The graph includes data from all population (or samples of τ_2) as well as the mean value.

It is not always possible to establish a mathematical correlation between two discrete points when there is no intersection between them. When there is no curve or equation to define the correlation between two variables or input factors, it becomes difficult to relate them. In such cases, isolines are a powerful tool in resolving these issues. Note that three graphs (Figures S6a, S6b, and S6c) are necessary to describe the relationship between H_{PI}, L_N, and Crops time.

Figures S6e and S6f display H_{PI} and L_N vs Crops time treatments τ_1- τ_4. The data is based on average values and shows a sigmoidal relationship, as mentioned previously. It is evident that the plants in treatment E1 exhibit greater height and leaf numbers over time compared to those in treatment E2. Even in Figure S6f, τ_1 achieves higher L_N values than τ_2, despite τ_2 having been administered with ON and having a greater H_{PI}.

Upon closer examination of Figure S6g, it becomes clear that the L_N trend between treatments τ_1 -τ_2 and τ_3-τ_4 suggests that treatments without ON result in higher L_N values with lower H_{PI}, until reaching a point where H_{PI} and L_N are equal. After this inflection point, the trend indicates that L_N in τ_1 remains more consistent than in τ_2, with τ_2 ultimately reaching a greater height and a larger number of leaves. Additionally, between treatments τ_3-τ_4, after the point of equality between L_N and H_{PI}, τ_4 experiences an increase in height while L_N remains relatively constant. On the other hand, τ_3 shows minimal growth in height and mostly in L_N.

Given that H_{Pl} and L_N are related to Crops time, and bearing in mind the opposite trends depicted in Figures S6e and S6f compared to Figure S6g; how can we reconcile this apparent contradiction? The use of 3D graphics with ISO lines may provide a solution to this, as we will further explore. Furthermore, it is important to recognize that the agronomic interpretation of the sample and the relationship between output variables and their causes may be incomplete.

In Figures S6g and S6h, treatments τ_1 and τ_2 show that after a certain point, the taller the plant, the more leaves and branches it has, respectively. However, in treatment τ_2, this trend is altered when analyzed in 3D (refer to Figure 5b in the manuscript).

In Figures S6g and S6h, a lower height in $\tau_3$compared to τ_4 is associated with a higher L_N and B_N, respectively. However, the trend for τ_4 has changed (refer to Figure 5d in the manuscript).

Figures S6l and S6j display the relationship between SS_N and H_{Pl}, as well as Crops time, respectively. In Figure S6l, all SS_N treatments demonstrate a statistically normal distribution in relation to height, and the analysis indicates that SS_N increases at higher H_{Pl}. When isolines are included, the sample still shows statistical normality, but the relationship with L_N varies.

Figure S6j illustrates the relationship between SS_N and the duration of Crops time. For the E2 treatments, it is clear that the growth profile is exponential for the entire sample.

In Figure S6k, the potential growth profile remains the same, and the ratio IN/LN is shown to be 1/2. Figure S6l demonstrates a normal pattern of stem growth over time, indicating that as the moment of inflorescence and fruiting approaches, the stem diameter decreases. Treatments without ON reach maximum thickness in less time than treatments with ON, suggesting that the plant is preparing for inflorescence, and the type and amount of nutrients interacting with the environment contribute to this effect. The analysis using isolines complements and enhances the understanding of vegetative growth and the relationship between output variables, resolving contradictory or incomplete elements identified through the morphometric analysis with 2D graphs and mathematical regressions.

Table S7. Multiple comparation of Tukey

τ_i	Ww		WD		HPl		LN		IN		BN		SD		NNS	
	M	TG	M	TG	M	TG	M	TG	M	TG	M	TG	M	TG	M	TG
τ_1	4.40	a	8.62	a	5.05	A	53.2	a	26.6	a	27.3	ab	0.73	A	-0.6	c
τ_2	4.57	a	9.68	a	5.13	A	50.6	a	25.3	a	30.4	a	0.80	A	0.2	c
τ_3	3.31	c	2.92	c	4.08	B	34.4	b	17.2	b	22.6	b	0.21	B	2.8	b
τ_4	3.95	b	5.10	b	4.42	C	37.0	b	18.5	b	23.0	b	0.32	B	4.6	a

Table S8. Sample Variance Analysis

τ_i	Sample variance									
	**Ww**	**WD**	**Chc1**	**Chc2**	**HPl**	**LN**	**IN**	**BN**	**SD**	**SSN**
**O**	1212.6	173.0	55.2	10.6	2628.5	105.2	26.3	37.0	0.8	43.3
τ_1	293.7	39.4	6.7	12.2	547.7	77.5	19.4	14.5	0.5	2.7
τ_2	1107.1	101.9	4.5	7.8	764.5	19.6	4.9	35.6	1.1	5.8
τ_3	44.8	6.0	19.1	9.8	82.5	28.3	7.1	42.9	0.0	10.3
τ_4	217.7	10.8	76.5	14.1	206.7	31.3	7.8	21.3	0.3	36.2

Figure S7. Histograms, Pearson´s lineal correlation coeficient, and statistical significance

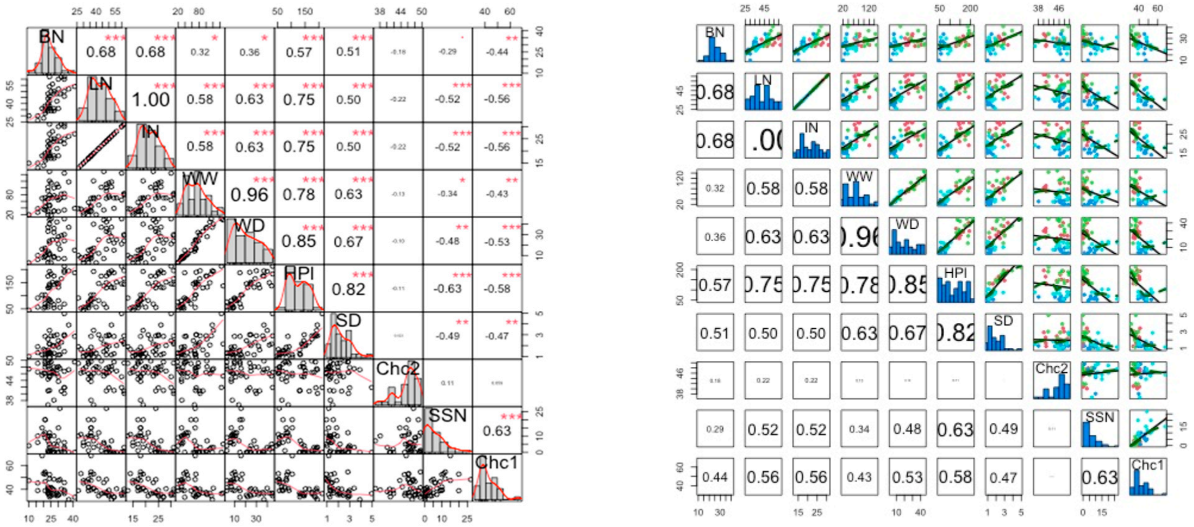

Height Plant (HPl), Number of leafs (LN), Internodes number (IN), Branches number (BN), Stem diameter (SD), Secondary stems number (SSN), Wet weight (WW), Dry weight (WD), Chlorophyll content (Chc), Chlorophyll content (Chc1), Chlorophyll content (Chc2) (τ_1, τ_2, τ_3, τ_4)

Note: The Wet Weight is sinonimous of Fresh Weight

Figure S8. Iguala Experimental Field &Taxco Experimental Field

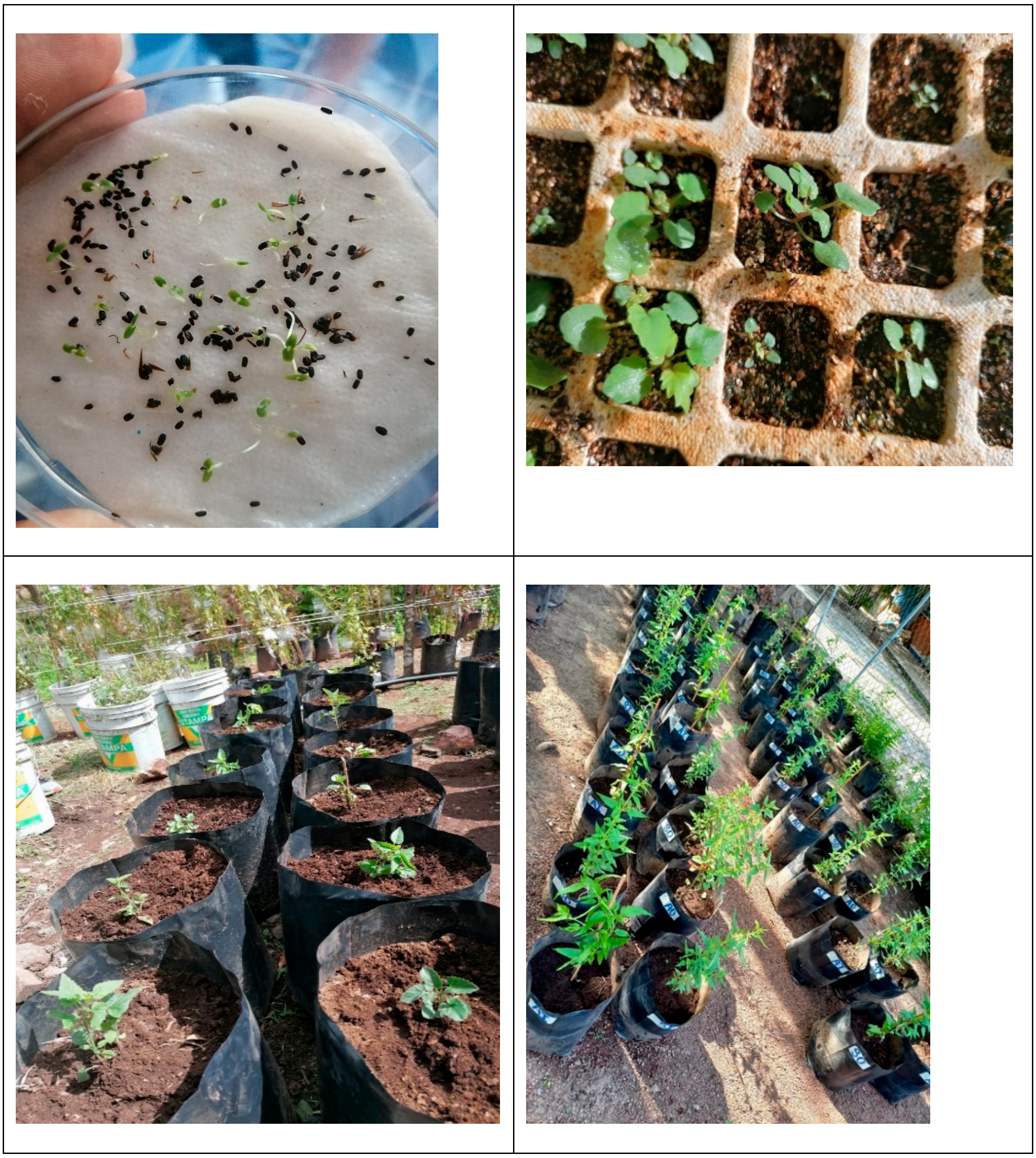

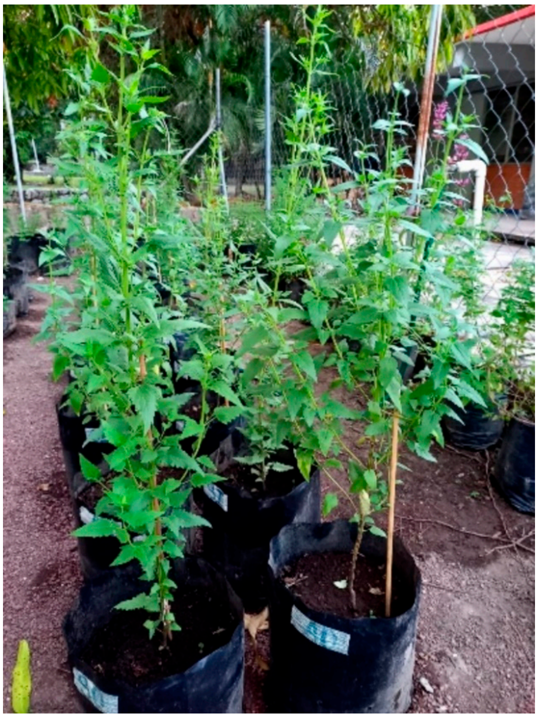

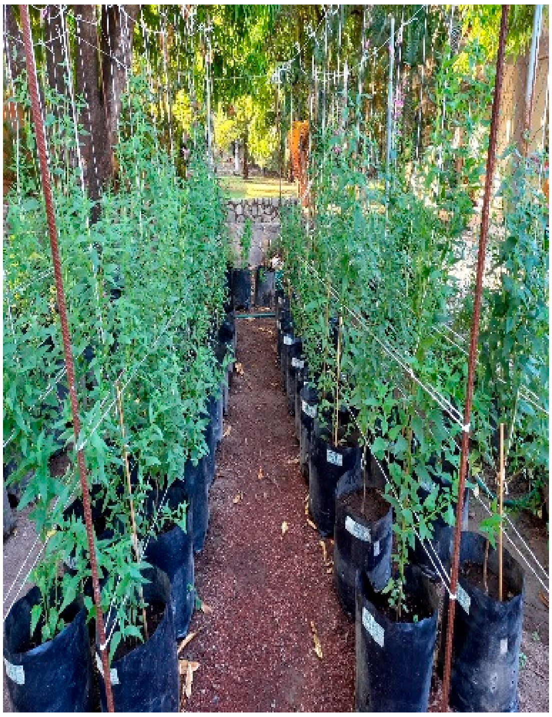

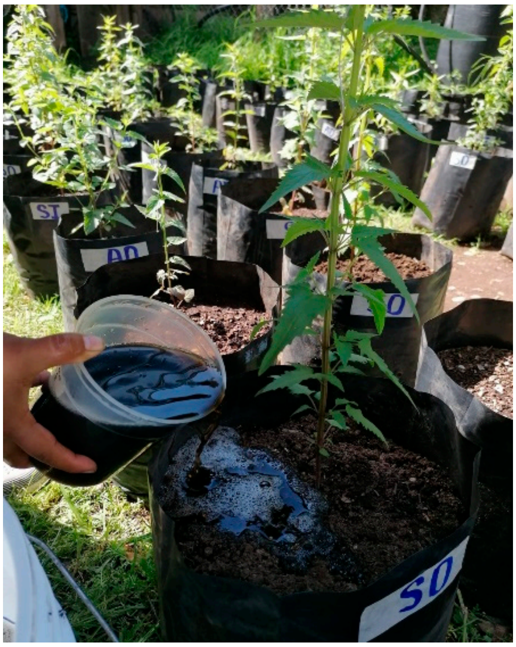
ST
AD
SO
SO

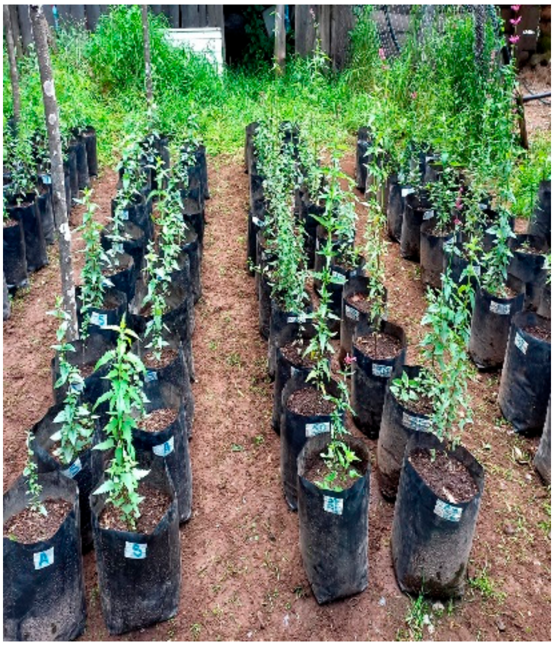
A
S

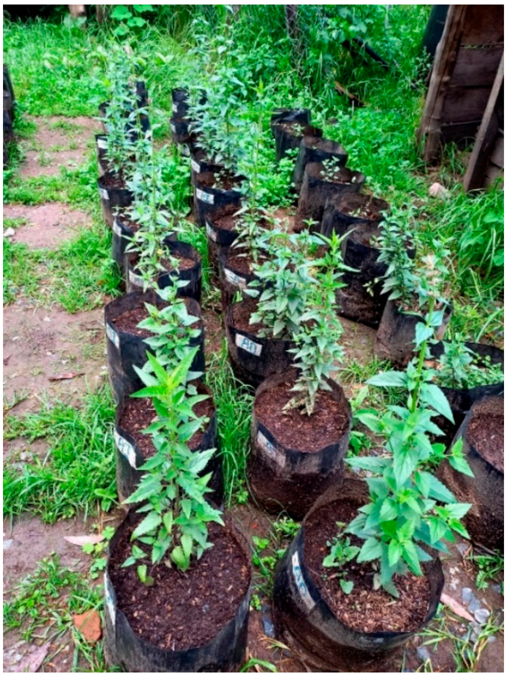

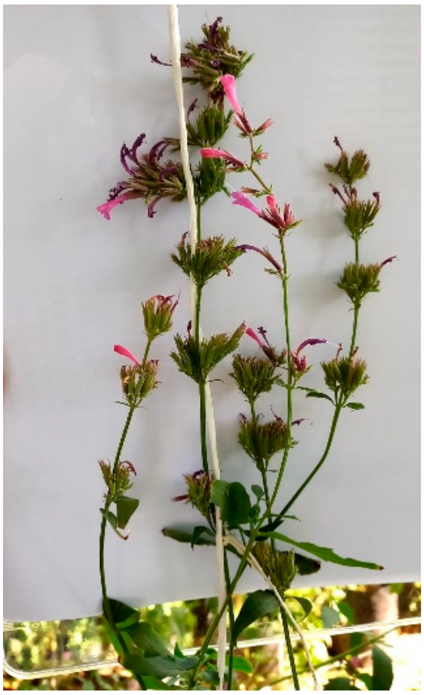

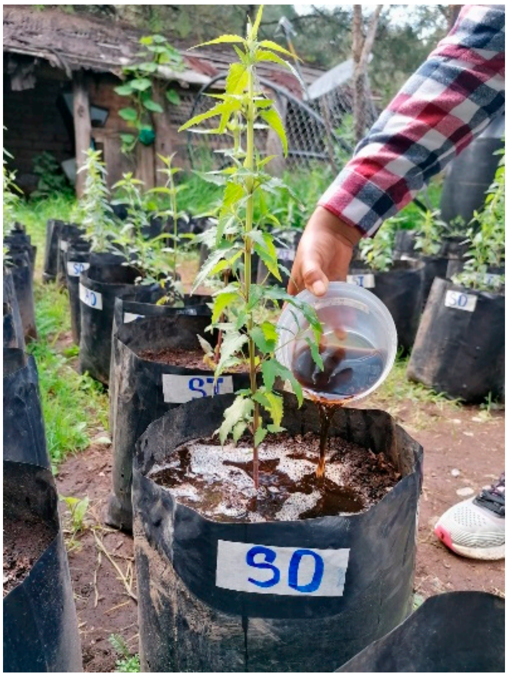
SO

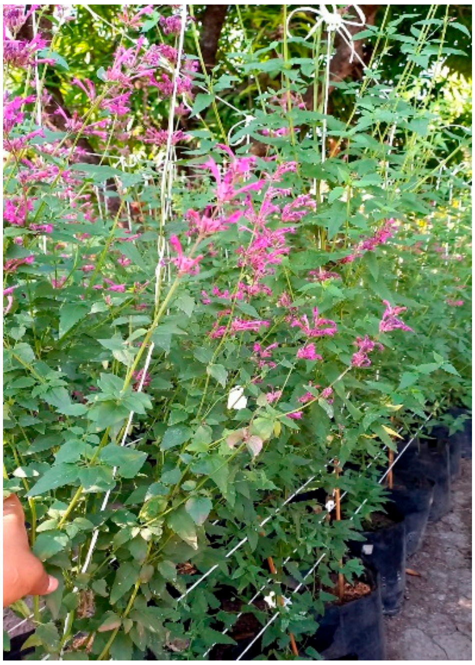

In order to make know information about BCSR approach, we include the following:

The BCSR approach has led to a significant divide between researchers and the consultant and farming communities. Despite farmers and consultants expressing confidence in the approach to managing soil health, the scientific community remains highly skeptical and lacks empirical evidence to support the notion that BCSR can enhance crop yields. The effectiveness of this approach in improving crop yields is still a topic of controversy due to insufficient data. [19]
Soil balancing consultants commonly believe that the availability and absorption of many nutrients or minerals in soil are affected by the presence or absence of other nutrients. By adding a nutrient, a shortfall or excess of another nutrient can be addressed.
Farmers conducted studies that revealed that over a period of 20 years, the Ca/Mg ratio in soil increased from approximately 1.25/1 to 4/1, while the CEC increased from around 14 to 25. The farmers explained that an increase in Mg levels results in an increased need for N by plants, which is one of the reasons why the Ca/Mg ratio is important. Adding calcium to the soil supports soil aggregation since calcium has a positive charge and helps negatively charged clay particles stick together. [45]

Table S9 compares two types of soil from the same area that were studied over a 20-year period. One soil underwent BCSR approach practices, while the other did not. The study shows that in the soil without BCSR practices, the presence of Na (sodium) makes it highly saline. However, in the soil with BCSR practices, Ca (calcium) and Mg (magnesium) can replace Na, resulting in less saline soil.
Calcium has the ability to form bonding bridges with clay, which results in increased porosity and prevents the leaching of nutrients from organic matter. At the University of Missouri, an interesting Ca/Mg relationship with Water-Stable Aggregation was discovered. Soil aggregation is the process of binding several soil particles into secondary units, and soil aggregates, especially water-stable aggregates, are particularly important for good soil structure and high-water infiltration. These properties are crucial in determining soil quality and directly influence soil and water conservation. Water-stable aggregation is often used as a measure of soil structure and is a suitable index of soil resistance to dispersion and compaction. Aggregate stability directly influences plant emergence, water infiltration, and soil erosion. [52]

Table S9. Comparative test between two soils in the same area. [45]

Element	NT	WT
% Ca	42.2	67.9
% Mg	33.9	16.7
% K	3.1	5.0
% Na	8.8	1.5
Ca/Mg	1.24	4.07
CEC	14.75	24.03

NT=No treatment, WT= with Treatment.

Table S10 displays the levels of water-stable soil aggregates based on the ratio of Ca/Mg. When Ca^{+2} base saturation increases and Mg^{+2} base saturation decreases, the levels of water-stable soil aggregates also increase. The highest levels of water-stable soil aggregates were achieved at Ca/Mg ratios ranging from 4.2/1 to 6.1/1. After that, the increase in Ca^{+2} base saturation became the main factor responsible for the highest levels of water-stable soil aggregates.

Table S10. Values of water stable soil aggregate depending on Ca/Mg ratio. [45]

Ca^{+2} Base Saturation %	Mg^{+2} Base Saturation %	Ca/Mg ratio	Water stable soil Aggregate %
70.0	11.5	6.1/1	40
61.5	14.7	4.2/1	40
53.0	16.0	3.3/1	36
41.0	36.0	1.1/1	33

Summary and generalities

In short, this research contributes to establishing:

a) The graphics created using MATLAB illustrate how temperature and relative humidity can impact different output factors. These visuals can help identify relationships in plant growth and development, and play a crucial role in making decisions related to agricultural management, including pruning, fertilization, crop rotation, and tillage techniques.
b) The plants in E2 have been almost left after being harvested in order to observe their natural response to the environment. This observation has revealed that the plants are capable of surviving with minimal human intervention, which is crucial for reintroducing the species into its natural habitat. This is especially important as the population of the species has declined due to human activities and climate change. This also means that BCSR soil and BCSR soil + ON treatments are capable to produce fruits after first vegetative growth cycle and harvest.
c) Plants in E1 did not survive after harvest, even with irrigation. This information helps us make decisions about the timing of dry matter and seed harvest, both in natural conditions and in E2.
d) The theoretical analysis presented in the manuscript provides scientific evidence to classify *Agastache mexicana subsp. mexicana* as a Type C3 plant. It also supports the inclusion of shade as a factor in experiments and as a key element for plant survival.
e) Here, the research contains important information previously unreported on CEC content, and balanced percentages of Ca^{2+}, Mg^{2+}, and K^{+} in loam soils. These findings align with the soil BCSR approach used in our study to make decisions about ON application into mountain.

f) It is necessary to measure secondary metabolites content in leaves due to chilling, frost, heat, and salinity stress and the defense mechanisms for changes in the production of secondary metabolites.
